# MHC class II *DQB* diversity in the Japanese black bear, *Ursus thibetanus japonicus*

**DOI:** 10.1186/1471-2148-12-230

**Published:** 2012-11-29

**Authors:** Yoshiki Yasukochi, Toshifumi Kurosaki, Masaaki Yoneda, Hiroko Koike, Yoko Satta

**Affiliations:** 1 Department of Evolutionary Studies of Biosystems, the Graduate University for Advanced Studies (SOKENDAI), Shonan Village, Hayama, Kanagawa 240-0193, Japan; 2 Japan Wildlife Research Center, 3-10-10 Shitaya, Taitou-ku, Tokyo 110-8676, Japan; 3 The Kyushu University Museum, Kyushu University, 6-10-1 Hakozaki, Higashi-ku, Fukuoka city 812-8581, Japan

**Keywords:** Balancing selection, Conservation genetics, *DQB*, Genetic diversity, Japanese black bear, Major histocompatibility complex, Ursidae, *Ursus thibetanus*

## Abstract

**Background:**

The major histocompatibility complex (MHC) genes are one of the most important genetic systems in the vertebrate immune response. The diversity of MHC genes may directly influence the survival of individuals against infectious disease. However, there has been no investigation of MHC diversity in the Asiatic black bear (*Ursus thibetanus*). Here, we analyzed 270-bp nucleotide sequences of the entire exon 2 region of the MHC *DQB* gene by using 188 samples from the Japanese black bear (*Ursus thibetanus japonicus*) from 12 local populations.

**Results:**

Among 185 of 188 samples, we identified 44 MHC variants that encoded 31 different amino acid sequences (allotypes) and one putative pseudogene. The phylogenetic analysis suggests that MHC variants detected from the Japanese black bear are derived from the *DQB* locus. One of the 31 DQB allotypes, Urth-DQB*01, was found to be common to all local populations. Moreover, this allotype was shared between the black bear on the Asian continent and the Japanese black bear, suggesting that Urth-DQB*01 might have been maintained in the ancestral black bear population for at least 300,000 years. Our findings, from calculating the ratio of non-synonymous to synonymous substitutions, indicate that balancing selection has maintained genetic variation of peptide-binding residues at the *DQB* locus of the Japanese black bear. From examination of genotype frequencies among local populations, we observed a considerably lower level of observed heterozygosity than expected.

**Conclusions:**

The low level of observed heterozygosity suggests that genetic drift reduced *DQB* diversity in the Japanese black bear due to a bottleneck event at the population or species level. The decline of *DQB* diversity might have been accelerated by the loss of rare variants that have been maintained by negative frequency-dependent selection. Nevertheless, *DQB* diversity of the black bear appears to be relatively high compared with some other endangered mammalian species. This result suggests that the Japanese black bears may also retain more potential resistance against pathogens than other endangered mammalian species. To prevent further decline of potential resistance against pathogens, a conservation policy for the Japanese black bear should be designed to maintain MHC rare variants in each local population.

## Background

In Japan, the Asiatic black bear (*Ursus thibetanus*) is classified as the subspecies (*Ursus thibetanus japonicus*). The Japanese black bear currently inhabits two main islands in Japan, Honshu and Shikoku. Historically, these bears were also distributed in Kyushu, but no native bears have been captured there for several decades [1,2]. However, there have been several reports of sightings of an animal believed to be a black bear in Kyushu [3]. Research on the genetic diversity in the black bear has begun in recent years and has been developed with the use of genetic markers such as mitochondrial DNA (mtDNA) sequences and microsatellites [4-12]. Using mtDNA markers we have found that the Japanese black bear is genetically quite distinct from other Asiatic black bears [8]. Sequences of the mtDNA control region indicated that the Japanese black bear could be divided genetically into three groups: western, eastern, and Shikoku/Kii-hanto populations [7,8]. Analyses of mtDNA and microsatellite markers [6,8] revealed lower genetic diversity and higher genetic differentiation in the western population than in the other populations. In fact, in most of the management units in western Japan the species has been designated as “endangered” in the Red Data Book of Japan [13] because of the fragmentation and isolation of their habitats.

The major histocompatibility complex (MHC) genes play an important role in the resistance to various pathogens, especially in wild animals. If MHC polymorphism declines, resistance to infectious diseases will decrease [14]. The MHC genes are thought to influence the survival of species or populations against pathogens. Therefore, MHC genetic variability in local populations is regarded as a potentially important index in conservation genetics.

In the human genome, the MHC region is located on the short arm of chromosome 6 at 6p21.3 and covers roughly 4 Mbp comprising 224 genes [15]. The human MHC region encodes at least three functionally and structurally different types of molecules, class I, class II, and class III. The MHC class II region is composed of five sub-regions (*DP*, *DQ*, *DR*, *DM*, and *DO*). Each sub-region contains at least one set of *A* and *B* genes, and these encode two polypeptide chains, *α* and *β* chains, respectively, which make up a heterodimeric molecule. For example, in a DQ molecule, the *DQA* gene encodes the *α-*chain, while *DQB* encodes the *β-*chain. The DP, DQ, and DR molecules are primarily important for the presentation of peptides and they are classified as classical class II molecules.

For the last three decades, studies of MHC allele variations have been performed not only in humans and laboratory animals but also in populations of wild mammals. Many such studies of wild mammals have demonstrated a relationship between low levels of MHC diversity and decreases in species population fitness (see below). MHC diversity was examined in pinniped species, which are believed to be closely related phylogenetically to ursine species [16,17]. Hoelzel and colleagues [18] reported that genetic variation at the *DQB* locus in the southern elephant seal (*Mirounga leonina*) did not appear to be low, whereas the northern elephant seal (*Mirounga angustirostris*), which was thought to have experienced a severe population bottleneck, exhibited much less variation at this locus. Lento et al. [19] and Weber et al. [20] demonstrated low genetic diversities at the MHC loci in the New Zealand sea lion (*Phocarctos hookeri*), and the northern elephant seal. The census sizes of these pinniped species have also diminished in the recent past probably due to human activity. In the case of non-carnivore mammals, heterozygosity of the *DRB* gene in a population of the striped mouse (*Rhabdomys pumilio*), has been shown to influence infection status [21]. A low level of diversity at the *DAB* locus (an MHC class II gene in marsupials) in the western barred bandicoot (*Perameles bougainville*) may influence its vulnerability against novel pathogens [22].

Many studies have revealed a correlation between MHC diversity and resistance against infectious disease; however, others have demonstrated that this is not the case universally. For example, genetic variation in *DQ* and *DR* sequences in California sea lions (*Zalophus californianus*), which is a thriving species, is lower than that in several other mammalian species [23,24]. The Tasmanian devil (*Sarcophilus harrisii*), which is an endangered species, has low MHC diversity, but individuals with restricted MHC repertoires (These individuals possessing only one of two phylogenetic groups) may be resistant to the devil facial tumor disease [25]. However, their MHC variants were not assigned to loci because of a lack of genomic information. Thereafter, the research group has shown that there is no direct evidence that MHC genetic difference between tumor-free and infected animals is associated with the resistance against the infection although certain genotypes at two MHC-linked microsatellite markers seem to be more frequently detected from healthy animals [26]. The *DRB* locus in the mountain goat (*Oreamnos americanus*) exhibited low genetic diversity, but this does not appear to have affected the resistance to infectious diseases in this species [27].

For Ursidae, studies of MHC diversity are somewhat limited. In the giant panda (*Ailuropoda melanoleuca*), MHC studies are relatively advanced and have been used to propose some possible implications for captive management [28-34]. Low genetic diversity at the *AimeDRB* and *AimeDQA* loci has been observed in the giant panda [28,30]. Wan et al. [31] attempted to understand the genomic structure and evolutionary history of *Aime*-MHC genes. The study has showed that the giant panda possesses a single *DYB*-like pseudogene (the DY subregion was thought to be ruminant-specific MHC genes, but may be shared by members of Laurasiatheria superorder.). Chen et al. [34] examined genetic diversity at seven *Aime*-MHC class II genes from 121 individuals and showed the presence of two monomorphic and five polymorphic loci. In brown bears (*Ursus arctos*) three *DQA* variants and 19 *DRB* variants were identified in 32 and 38 bears, respectively, although loci have not been assigned [35,36]. Kuduk and colleagues reported the expression of three MHC class I, two DRB, two DQB and one DQA loci in the brown bear [37]. Other than these studies on the giant panda and brown bear, there have been no published reports on the MHC genetic diversity in the Ursidae.

Although the direct contribution of MHC genes to the immunological fitness of animals is still unclear, the relatively high genetic diversity of the MHC reflects the abundance in repertoire of binding peptides, derived from pathogens. We thus believe that MHC diversity potentially contributes to resistance against environmental pathogens and species survival, indicating that study of these loci is essential for designing a plan for conservation management of animals. Because of large variability of MHC, the examination of MHC diversity is also useful for the management of endangered wild-animal populations that show no variation in genetic markers such as microsatellite loci [38]. In our previous study, we reported a functional *DQB* sequence (1,150 bp) in the Japanese black bear, identified using the rapid amplification of cDNA ends method [39]. Here, we investigated polymorphism in exon 2 of *DQB* that contains the hypervariable region involved in binding antigens for the Japanese black bear to consider the potential implications for resistance against pathogens and estimate the status of local populations on the basis of their MHC variation.

## Results

### Features of the *DQB* exon 2 variants in the Japanese black bear

**Figure 1:**
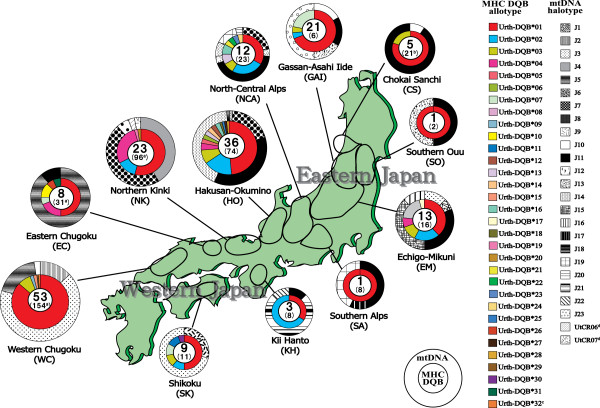
**Geographical distribution of DQB allotypes and haplotypes of mtDNA control region.** The inner pie chart indicates the observed frequencies of DQB allotypes in the Japanese black bear. The outer pie chart indicates and the observed haplotype frequencies of the mtDNA control region [8]. Twelve local populations based on the conservation and management units for the Japanese black bear [42] are shown as bold circles. The number of samples for DQB allotypes from each location is depicted in the center of each corresponding chart, and the number in parenthesis indicates the sample size used for mtDNA analysis of our previous study [8]. Superscript a represents the number of samples analyzed by Ishibashi and Saitoh [5] in addition to our previous study [8]. Superscript b indicates the number was obtained by identification of individuals using microsatellite analysis [90]. Superscript c indicates the putative pseudogene. Superscript d indicates haplotypes of mtDNA identified by Ishibashi and Saitoh [5].

**Figure 2:**
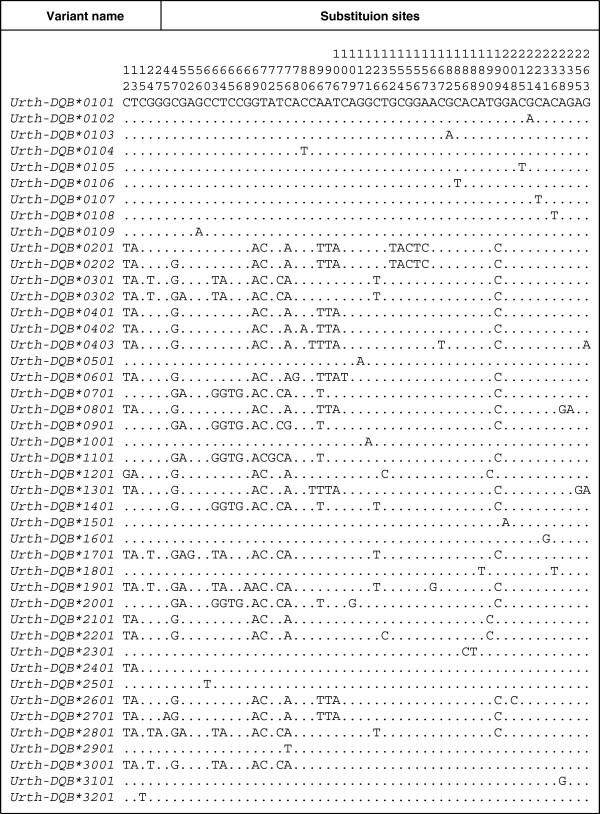
**Alignment of segregating sites among the *DQB *exon 2 variants in the Japanese black bear.** Dots indicate identity with the nucleotides of Urth-DQB*0101. The first two numeric characters after an asterisk in a variant name represent an allotype

The entire exon 2 region of the *DQB* locus (270 bp) was determined for the Japanese black bear. The amplified region corresponded to amino acids 6 to 94 of the *β* chain of the human DR molecule [40,41]. The frequency of variant typing success was lowest for tooth samples (Ca. 54%), and was highest for blood (Ca. 92%). This result probably reflects the difference in the amount of gDNA in each sample type. From 188 bear samples in 12 of 19 conservation and management units designed by Yoneda [42] (Figure 1), we detected 47 *DQB* variants. However, the application of criteria for genotyping used in this study (see Methods) reduced the number to 44 variants [DDBJ: AB634514–AB634536] and to 185 bear samples (Figures 1, 2 and Table 1). In the present study, no more than two different variants per individual were detected from the clones of the PCR products.

**Table 1:** **The observed number of nucleotide *DQB *variants detected from each conservation and management unit**

**Conservation and management unit**	**CS**	**GAI**	**SO**	**EM**	**SA**	**NCA**	**HO**	**NK**	**EC**	**WC**	**KH**	**SK**	**Total**
No. of sample	5	21	1	13	1	12	36	23	8	53	3	9	**185**
No. of variant	2	4	1	7	1	10	18	8	7	10	3	6	
Urth-DQB*0101	8	29	2	10		8	34	23	6	86	2	9	**217**
Urth-DQB*0102										2			**2**
Urth-DQB*0103					2								**2**
Urth-DQB*0104									2				**2**
Urth-DQB*0105								2					**2**
Urth-DQB*0106										2			**2**
Urth-DQB*0107										1			**1**
Urth-DQB*0108													**1**
Urth-DQB*0109										1			**1**
Urth-DQB*0201		2		5		5	4	2			2	2	**22**
Urth-DQB*0202						1	8	4			2		**15**
Urth-DQB*0301				1		2	1			8		2	**14**
Urth-DQB*0302	2	2		2			5						**11**
Urth-DQB*0401				2			2	12	3				**19**
Urth-DQB*0402								1					**1**
Urth-DQB*0403							1						**1**
Urth-DQB*0501							2						**2**
Urth-DQB*0601							2						**2**
Urth-DQB*0701		9		5		2			1			2	**19**
Urth-DQB*0801						2							**2**
Urth-DQB*0901										2			**2**
Urth-DQB*1001									2				**2**
Urth-DQB*1101												2	**2**
Urth-DQB*1201							2						**2**
Urth-DQB*1301							2						**2**
Urth-DQB*1401							2						**2**
Urth-DQB*1501										2			**2**
Urth-DQB*1601							2						**2**
Urth-DQB*1701				1									**1**
Urth-DQB*1801							1						**1**
Urth-DQB*1901								1					**1**
Urth-DQB*2001								1					**1**
Urth-DQB*2101						1							**1**
Urth-DQB*2201						1							**1**
Urth-DQB*2301						1							**1**
Urth-DQB*2401						1							**1**
Urth-DQB*2501							1						**1**
Urth-DQB*2601									1				**1**
Urth-DQB*2701							1						**1**
Urth-DQB*2801							1						**1**
Urth-DQB*2901										1			**1**
Urth-DQB*3001												1	**1**
Urth-DQB*3101									1				**1**
Urth-DQB*3201										1			**1**

CS: Chokai Sanchi; GAI: Gassan-Asahi Iide; SO: Southern Ouu; EM: Echigo-Mikuni; SA: Southern Alps; NCA: North-Central Alps; HO: Hakusan-Okumino; NK: Northern Kinki; EC: Eastern Chugoku; WC: Western Chugoku; KH: Kii Hanto; SK: Shikoku.

**Figure 3:**
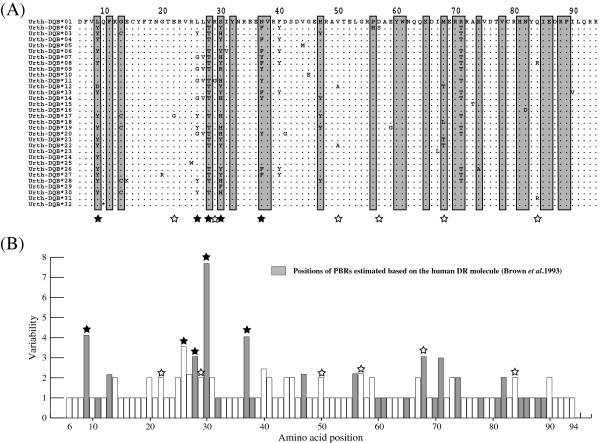
**Amino acid sequences and the variability in the exon 2 of bear *DQB*s.** (**A** - **B**) Gray boxes indicate positions of the putative peptide binding residues (PBRs) deduced from the amino acid position in the human DR molecule, and the numbers across the sequence correspond to amino acid positions based on the chain of the human DR structure [40]. A filled asterisk represents the putative site under balancing selection identified by both of PAML and OmegaMap programs, while an open asterisk is that by the OmegaMap only. (**A**) Alignment of the predicted amino acid sequence translated from nucleotides of MHC *DQB* variants in the Japanese black bear. Dots indicate identity with amino acids of Urth-DQB*01. Urth-DQB*32 includes the putative premature stop codon within the sequence. (**B**) The variability level at amino acid residues among *DQB* variants of the Japanese black bear. The variability level was estimated by a Wu-Kabat plot [85]

**Figure 4:**
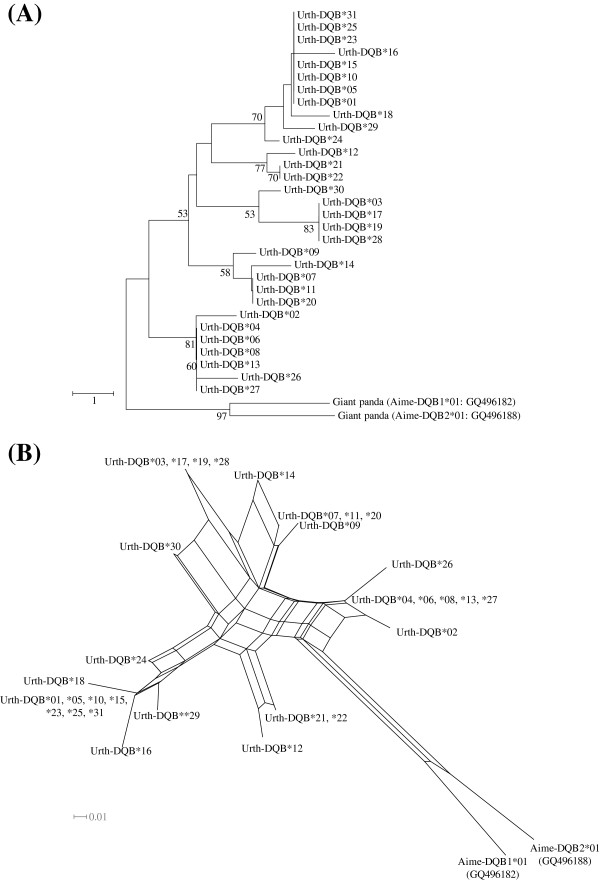
**Neighbor-joining tree and network based on amino acids in the PBR of *DQB *allotypes.** (**A**) A neighbor-joining (NJ) tree was constructed by using the number of differences method. *DQB* sequences of the giant panda are used as outgroups. Only bootstrap values over 50% are shown in this figure. (**B**) Neighbor-Net network is also based on the sequences of NJ tree

Thirty-one distinct amino acid sequences (Urth-DQB*01 to Urth-DQB*31) were translated from nucleotide sequences of the 43 variants (Urth-DQB*0101 to Urth-DQB*3101) (Figure 3). Because one variant examined (Urth-DQB*3201) included a premature stop codon in the sequence, it was recognized as a pseudogene (Figure 3A). This Urth-DQB*3201 was detected from one individual which was heterozygous with this variant and Urth-DQB*0101. An NCBI BLAST search showed that sequence similarities between bear *Urth-DQB* and panda *Aime-DQB* variants were 84% to 91% and 93% to 96% similarity at the amino acid and nucleotide levels, respectively [34]. Of all the variants, Urth-DQB*0101, *0104, *0401, *1001, and *2601 correspond to the Urth-DQB*a to Urth-DQB*e variants detected in our previous study [39], at the DNA level. Thus, in the present study we additionally identified 39 novel variants at the DNA level in 178 samples. The nucleotide sequence of Urth-DQB*0101 was identical to that found in exon 2 of a cDNA sequence derived from mRNA of a bear sample in our previous study [39]. This suggests the amplification of a functional *DQB* locus in the present study, although there is no evidence of protein translation. Deletions and insertions were not detected among the sequences. There were 58 segregating sites among the 44 different nucleotide sequences (Figure 2). Amino acid substitutions were observed at 30 sites among the 31 allotypes (Figure 3). While 11 of these substituted sites were located in the putative peptide-binding residues (PBRs) [40], 19 corresponded to non-PBR positions. The presence of more substitution sites in the non-PBRs appears to suggest a loss of the open reading frame in the bear *DQB* gene. However, there were no frameshift or nonsense mutations in *Urth-DQB* sequences with the exception of the Urth-DQB*3201 although our study was limited to exon 2 (Figure 3). In addition, amino acids at 8 of 12 PBR codons that showed a monomorphic pattern were also conserved in the human *DQB1* locus, and most of the segregating sites at the non-PBRs among bear *DQB* variants were singletons. Although we cannot completely exclude the possibility that pseudogenes are present in our data set, the fact that our results show more substitution sites in the non-PBRs than PBRs indicates that it is unlikely that we amplified pseudogenes. Therefore, we defined all bear *DQB* variants as the putative functional DQB molecule with the exception of Urth-DQB*3201. Thirty-one DQB allotypes could be classified into 15 types of PBR (i.e., 15 different amino acid sequences of the PBRs; Figure 4). In 184 bears (excluding the individual with the putative pseudogene Urth-DQB*3201), 57 of 64 heterozygotes possessed a combination of different types of PBR sequences.

On the basis of the nucleotide sequences of the black bear *DQB* variants, we performed a search for intragenic recombination by using the GENECONV program [43]. Permutation testing indicated the possibility of recombination between some pairs of variants (Urth-DQB*0101, Urth-DQB*0104, Urth-DQB*0109, Urth-DQB*0402, Urth-DQB*0601, Urth-DQB*0701, Urth-DQB*0901, Urth-DQB*1101, Urth-DQB*1301, Urth-DQB*2001, Urth-DQB*2501, Urth-DQB*2701, Urth-DQB*2901, Urth-DQB*3001, Urth-DQB*3101, and Urth-DQB*3201). However, the Bonferroni-corrected BLAST-like test in GENECONV indicated no significant values (*P* = 1.0) for recombination events. In addition, not only the method described by Satta [44] but also the RDP4 program [45] indicated no significant signals for recombination or gene conversion. Thus, we used all the *DQB* variants of the Japanese black bear for further analyses.

### Gene tree based on exon 2 sequences of ursine MHC class II genes

**Figure 5:**
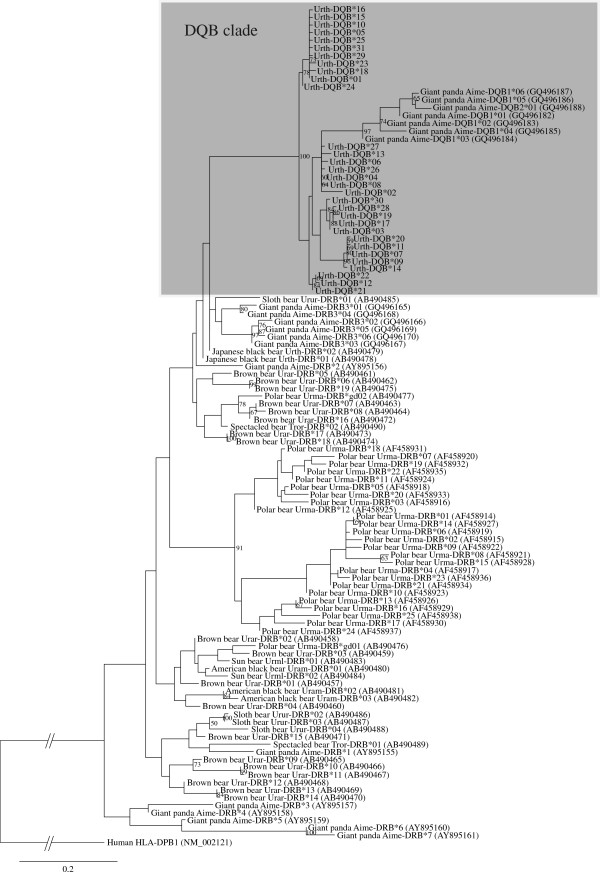
**Maximum likelihood tree for the exon 2 region of MHC class II genes.** This tree is constructed based on 79 amino acids in the MHC class II *DRB* and *DQB* exon 2 region. The genetic distances are computed by the JTT model with gamma distribution. Only bootstrap values over 50% are shown in this figure. Numbers in parentheses are Genbank accession numbers

A maximum likelihood (ML) tree based on amino acid sequences of partial DQB and DRB exon 2 from the Ursidae species was constructed (Figure 5). In this ML tree, the DQB sequences formed a clearly separated cluster from the DRB sequences, in agreement with Yasukochi et al. [39]. These trees showed that the 31 sequences from the Japanese black bear formed a monophyletic group with the DQBs of the giant panda. The Bayesian and Neighbor-joining (NJ) [46] trees also showed a monophyletic clade of the black bear DQBs and panda DQBs (see Additional File 1: Figure S1).

In the ML tree, DRBs of some ursine species were mixed with each other although the orthology of their sequences was unclear, indicating a possible trans-specific mode in the DRBs of ursine species. In the black bear/panda DQB clade, twenty black bear DQB allotypes (Urth-DQB*02 to *04, *06 to *09, *11 to *14, *17, *19 to *22, *26 to *28, and *30) appear to cluster more closely with panda rather than other black bear DQBs, suggesting the trans-species polymorphism. However, the bootstrap value is low (41%), and the pairwise distance among the twenty bears and other bear DQB variants showed closer relationships than those between black bear and panda DQBs. For example, the pairwise distances between the Urth-DQB*04 and other black bear DQBs ranged from 0.01 to 0.08 (mean value, 0.06), while those between the Urth-DQB*04 and panda DQBs ranged from 0.09 to 0.16 (mean value, 0.13). In addition, the Bayesian tree showed that DQB sequences of Japanese black bear formed a distinct clade from those of giant panda (see Additional File 1: Figure S1). Therefore, the allelic lineage of twenty black bear DQBs and panda DQBs may be different even though the ML tree appears to show the pattern of trans-species evolution.

### Selection of the *DQB* exon 2 region

We estimated the mean numbers of synonymous and nonsynonymous substitutions within the 267-bp region of *DQB* exon 2 variants in the Japanese black bear by using a modified Nei–Gojobori method [47] (Table 2). For the black bear, the ratio of nonsynonymous to synonymous substitutions (*d*_N_/*d*_S_) was approximately 3.83 in the PBRs, 0.63 in the non-PBRs, and 1.48 in the entire exon 2 region. Although three statistical tests did not indicate recombination among all the *DQB* variants, according to the BLAST-like test without the Bonferroni-correction in GENECONV program (see above), 16 variants were shown to have possibly undergone recombination or gene conversion. Recombination would change the *d*_N_/*d*_S _value, however, even if these possible recombinants were removed, ratios of *d*_N_/*d*_S _showed slightly higher values: 4.20 in the PBRs, 0.58 in the non-PBRs, and 1.52 in the entire exon 2 region. This shows that inclusion of the possible recombinants does not affect the result. To evaluate the *d*_N_/*d*_S _of the black bear, we compared them with those of other mammalian *DQB* or *DRB* loci (Table 2). In the family Ursidae, the *d*_N_/*d*_S _ratio within the PBRs of *DQB* in the black bear was higher than that of the giant panda *DQB*s, while it was lower than that of brown bear *DRB*s (but these *DRB* variants are derived from at least two loci). Compared with other mammals, the *d*_N_/*d*_S _value of the black bear did not appear to be low. In addition, by using the PAML program with models that describe various selection pressures (M0 to M8) [48-50], ratios of *ω* were estimated for black bears (Table 3). Values of *ω* in the black bear were significantly higher than unity (log likelihood ratio test) under M2a and M8 models that allow for balancing selection (*ω*_2_ = 7.0 in M2a and *ω* = 5.7 in M8). The likelihood ratio test of hypotheses (M2a vs M1a and M8 vs M7) showed statistically significant values (*P* < 0.001), suggesting a balancing selection model is a better fit to the data compared to other models. Thus, the present results indicate that exon 2 of *DQB* in the black bear has been a target of balancing selection. The Bayesian analysis also showed that five codons were potentially under balancing selection. The mean value of *ω*, estimated with Bayesian inference by using the OmegaMap program [51], was also larger than unity (*ω* = 1.8). This analysis also suggested that 11 codons were probably under balancing selection. Of these 11 codons, five were estimated by both the PAML and the OmegaMap programs. A majority of the selected sites (β9, β28, β30, and β37) were located in the predicted PBRs (Figure 3).

**Table 2:** **Comparison of non-synonymous (*d***_**N**_**) and synonymous (*d***_**S**_**) substitution rates at the *DQB *exon 2 region**

**Species**	**Gene**	**No. of sample**	**No. of variant**	**Length**	**PBRs**				**Non-PBRs**				**Entire exon 2 region**						
					***d***_**N**_**/*d*x**_**S**_	***d***_**N**_			***d***_**N**_**/*d***_**S**_	***d***_**N**_			***d***_**N**_**/*d***_**S**_	***d***_**N**_			***d***_**S**_		
***Ursidae***																			
Japanese black bear^a^	*DQB*	184^*k*^	43^*k*^	267 bp	3.83	0.11	±	0.04	0.63	0.02	±	0.01	1.48	0.04	±	0.01	0.03	±	0.01
Giant panda^b^	*DQB1*	121	6	267 bp	1.33	0.02	±	0.06	0.47	0.01	±	0.02	0.71	0.02	±	0.05	0.01	±	0.03
Brown bear^*c^	*DRB*	38	19	267 bp	4.09	0.06	±	0.29	0.72	0.02	±	0.05	1.53	0.02	±	0.07	0.02	±	0.11
***Other carnivores***																			
African wild dog^d^	*DRB*	320	19	267 bp	5.28	0.06	±	0.21	0.56	0.01	±	0.02	1.72	0.02	±	0.04	0.02	±	0.07
Namibian cheetah^*e^	*DRB*	139	4	237 bp	2.01	0.06	±	0.25	0.37	0.01	±	0.05	0.76	0.04	±	0.13	0.02	±	0.10
European mink^f^	*DRB*	20	9	228 bp	1.78	0.06	±	0.16	0.26	0.01	±	0.02	0.65	0.03	±	0.09	0.02	±	0.06
***Non-carnivores***																			
Water buffalo^*g, h^	*DQB*	210	22	243 bp	2.65	0.07	±	0.37	0.92	0.03	±	0.13	1.34	0.03	±	0.14	0.03	±	0.19
Ma's night monkey^i^	*DQB1*	19	14	246 bp	2.71	0.05	±	0.19	0.95	0.02	±	0.07	1.39	0.02	±	0.07	0.02	±	0.10
Yellow necked mouse^j^	*DRB*	146	27	216 bp	7.75	0.09	±	0.38	1.13	0.02	±	0.06	2.46	0.02	±	0.05	0.02	±	0.12

The *d*_N _and *d*_S _are estimated by the modified Nei-Gojobori method.

^a^: present study; ^b^: Chen et al. [34]; ^c^: Goda et al. [36]; ^d^: Marsden et al. [91];

^e^: Castro-Prieto et al. [92]; ^f^: Becker et al. [93]; ^g^: Niranjan et al. [94]; ^h^: Sena et al. [95];

^i^: Diaz et al. [96]; ^j^: Meyer-Lucht and Sommer [97]; ^k^: An individual that has the putative pseudogene, Urth-DQB*3201, is excluded.

^*^: The number of MHC variants may be derived from multiple loci.

**Table 3:** **Inference of balancing selection for 43 black bear *DQB *variants**

**Method**	**Model code**	**Parameter estimates**	**Likelihood or confidential interval**	**Positively selected sites**
Maximum likelihood (PAML)	M0 (one ratio)	*ω* = 0.41	−1570	
	M1a (nearly neutral)	*p*_0_ = 0.85, *p*_1_ = 0.15	−1386	
	M2a (balancing selection)	*ω*_2_ = 7.00	−1326	β9^**^, β26^**^, β28^**^, β30^**^, β37^**^
	M3 (discrete)	*ω*_2_ = 7.98	−1325	
	M7 (beta)	*p* = 0.05, *q* = 0.07	−1394	
	M8 (beta and *ω*)	*ω* = 5.71	−1337	β9^**^, β26^**^, β28^**^, β30^**^, β37^**^
Bayesian inference (OmegaMap)	−	*ω* = 1.80^a^	*ω*: 0.30 - 12.49^a^ (95% HPD)	β9^**^, β22^**^, β26^**^, β28^*^, β29^**^, β30^**^, β37^*^, β50^*^, β57^*^, β68^*^, β84^*^

The putative pseudogene (Urth-DQB*3201) is excluded from this analysis. *ω* = *d*_N_ / *d*_S_. *p*_0_: the proportion of conserved sites with 0 <*ω* < 1. *p*_1_ (=1 - *p*_0_): the proportion of neutral sites. Values of *p* and *q* are parameters of the beta distribution. ^*^: posterior probability, *P* < 0.95; ^**^: posterior probability, *P* < 0.99. ^a^: a mean value. Bold characters indicate positions of the putative peptide binding residues (PBRs) deduced from the amino acid position in the human DR molecule [40].

### Estimation of the divergence time of *DQB* variants in the Japanese black bear

To estimate the divergence time of *DQB* variants in the Japanese black bear, we calculated the maximum genetic distance at synonymous sites that corresponded to the time to most recent common ancestor (TMRCA). The resulting value was 0.08 ± 0.03, and, by assuming 3.5 × 10^-9^ per site per year as the average synonymous substitution rate for mammalian nuclear DNA [52], the divergence time of the bear *DQB* variants was estimated to be about 11 million years ago (MYA). Even if the possible recombinants described above were removed, the result did not change.

### Geographical distribution and genetic variation in the MHC exon 2 variants in the conservation and management units

Five DQB allotypes (Urth-DQB*01, Urth-DQB*02, Urth-DQB*03, Urth-DQB*04, and Urth-DQB*07) were shared among the management units (Figure 1 and Table 1). In particular, Urth-DQB*01 was observed throughout all the management units. This allotype, Urth-DQB*01, corresponds to six nucleotide *DQB* variants (Urth-DQB*0101 to Urth-DQB*0109). The variant Urth-DQB*0101 was detected in a particularly large number (133) of bears. This suggests that Urth-DQB*01 may have been dominantly maintained in the black bear population in recent times. This is probably because of its important immune role against pathogens or for demographic reasons.

On the basis of the variant frequency in each of the six management units from which more than 10 individuals were sampled, the expected heterozygosity (*H*_e_) ranged from 0.33 to 0.85 (mean *H*_e_, 0.65) (Table 4). Values of observed heterozygosity (*H*_o_) ranged from 0.19 to 0.69 (mean *H*_o_, 0.42), which were lower than the *H*_e_ for all six units. This suggests non-random mating and/or population subdivision in each unit or the existence of null alleles. Among the six units, *H*_e_ and *H*_o_ were the highest in the North-Central Alps (NCA) unit (*H*_e_ = 0.85) and the Echigo-Mikuni (EM) unit (*H*_o_ = 0.69), respectively, while those of Western Chugoku (WC) were the lowest (*H*_e_ = 0.33, *H*_o_ = 0.19). In the NCA unit, the *H*_o_ value (*H*_o_ = 0.33) was significantly low (*P* < 0.001) compared with the highest *H*_e_ value (*H*_e_ = 0.85). The observed and expected heterozygosities of bears in western Japan (*H*_e_ = 0.54, *H*_o_ = 0.31) were lower than that of bears in eastern Japan (*H*_e_ = 0.73, *H*_o_ = 0.41). Nucleotide diversity (*π*_S_) based on synonymous substitutions within a unit ranged from 0.005 to 0.024 among the six units, and the overall value for black bears was 0.016. The *π*_S_ value of the WC unit (0.005) was the lowest among the six units.

**Table 4:** Gene diversities and nucleotide diversities for each conservation and management unit

		***MHC DQB *exon 2 region (267 bp)**					**mtDNA control region (Yasukochi *et al*. 2009)**			
	**Conservation and management unit**	**No. of samples**	**No. of alleles**	***H***_**e**_	***H***_**o**_	***π***_**S**_	**No. of samples**	**No. of haplotypes**	***h***	***π***
**Eastern Japan**	**Gassan-Asahi Iide (GAI)**	**21**	**4**	**0.48 ± 0.08**	**0.33**	**0.018 ± 0.009**	6	5	0.93 ± 0.12	0.011 ± 0.008
	**Echigo-Mikuni (EM)**	**13**	**7**	**0.79 ± 0.05**	**0.69**	**0.024 ± 0.011**	**16**	**7**	**0.82 ± 0.07**	**0.009 ± 0.006**
	**North-Central Alps (NCA)**	**12**	**10**	0.85 ± 0.05^*^	0.33^*^	**0.022 ± 0.010**	**23**	**8**	**0.85 ± 0.05**	**0.011 ± 0.007**^**d**^
	**Hakusan-Okumino (HO)**	**36**	**18**	0.76± 0.05^*^	0.39^*^	**0.019 ± 0.008**	**74**	**8**	**0.72 ± 0.03**	**0.010 ± 0.006**
**Western Japan**	**Northern Kinki (NK)**	**23**	**8**	**0.68 ± 0.06**	**0.56**	**0.016 ± 0.008**	**96 (64)**	**4**	**0.75 ± 0.03**	**0.011 ± 0.007**
	Eastern Chugoku (EC)	8	7	0.83±0.07^*^	0.38^*^	0.013 ± 0.007	**31 (22)**	**2**	**0.25 ± 0.10**	**0.003 ± 0.002**
	**Western Chugoku (WC)**	**53**	**11**	0.33 ± 0.06^*^	0.19^*^	**0.005± 0.003**	**154 (33)**	**6**	**0.56 ± 0.04**	**0.005 ± 0.004**
	Shikoku (SK)	9	6	0.73 ± 0.10^*^	0.33^*^	0.021 ± 0.010	**11**^**c**^	**2**	**0.55 ± 0.07**	**0.009 ± 0.006**
	**Overall**	**175**	**44**	0.65 ± 0.03^*^	0.36^*^	**0.016 ± 0.007**	**455**	**24**	**0.90 ± 0.01**	**0.013 ± 0.008**

Bold character indicates conservation and management units which have more than 10 samples. Chokai Sanchi, Southern Ouu, Southern Alps and Kii Hanto units are excluded due to small number of samples. *H*_e_: the expected heterozygosity; *H*_o_: the observed heterozygosity. *π*_S_ or *π*: the nucleotide diversity. *h*: the haplotype diversity. ^*^: a significant departure from Hardy-Weinberg equilibrium (*P* < 0.01). ( ): the number of haplotype data analyzed by [5]. ^a^: the number includes subhaplotypes. ^b^: the number was taken from the result of an identification of individuals using microsatellite analysis [90] because samples were obtained by hair trap. ^c^: the number is samples that are newly added to samples used in our previous study [8]. ^d^: the *π* value described in [8] (*π* = 0.001) was incorrect, and 0.01 was a correct value.

We calculated four statistics of genetic differentiation (*K*_ST_, *F*_ST_, Hedrick’s *G*’_ST_ and Jost’s *D*) for pairs of management units by using the nucleotide sequences of *DQB* variants (Table 5). Among the six management units, most *K*_ST _and *F*_ST _values indicated statistically significant levels of genetic differentiation, including those between the WC and other units (mean *K*_ST_, 0.12; mean *F*_ST_, 0.17), and the Gassan-Asahi Iide (GAI) and other units (mean *K*_ST_, 0.05; mean *F*_ST_, 0.08). With respect to Hedrick’s *G*’_ST_ and Jost’s *D*, these values trended to be high between the WC and other units. The high values of *G*’_ST_ and *D* (more than 0.05) and significant values of *K*_ST _and *F*_ST _were observed only between the WC and EM/NCA/Hakusan-Okumino (HO) units (mean *K*_ST_, 0.13; mean *F*_ST_, 0.20; mean *G*’_ST_, 0.12; mean *D*, 0.07). We calculated average values for each of four statistics between management unit pairs in western and eastern Japan. The results indicated that the values for western Japan (*K*_ST_ = 0.05, *F*_ST_ = 0.10, *G*’_ST_ = 0.05, *D* = 0.03) were higher than those for eastern Japan (*K*_ST_ = 0.02, *F*_ST_ = 0.05, *G*’_ST_ = 0.02, *D* = 0.01).

**Table 5:** **Four statistics of genetic differentiation for *DQB *variants between the pairs of management units**

	**Eastern Japan**					**Western Japan**		
**Conservation and management unit**		**Gassan-Asahi Iide (GAI)**	**Echigo-Mikuni (EM)**	**North-Central Alps (NCA)**	**Hakusan-Okumino (HO)**	**Northern Kinki (NK)**	Eastern Chugoku (EC)	**Western Chugoku (WC)**
**Eastern Japan**	**GAI**	*K*_ST_ / *F*_ST_/ Hedrick's *G*'_ST_/ Jost's *D*						
	**EM**	0.040^*^/ 0.068^*^/ 0.036^**^/ 0.022^**^						
	**NCA**	0.057^**^/ 0.112^**^/ 0.049^**^/ 0.030^**^	-0.006/ -0.012/ -0.022^**^/ -0.015^**^					
	**HO**	0.030^**^/ 0.063^**^/ 0.078^*^/ 0.049^*^	0.008/ 0.029^*^/ -0.002^*^/ -0.002^**^	-0.001/ 0.026^*^/ -0.013^**^/ -0.010^**^				
**Western Japan**	**NK**	0.057^**^/ 0.104^**^/ 0.063^*^/ 0.037^*^	0.017/ 0.054^*^/ 0.016^**^/ 0.011^**^	-0.002/ 0.069^**^/ 0.021/ 0.015^**^	-0.001/ 0.029^**^/ 0.023^**^/ 0.016^**^			
	EC	0.009/ 0.116^**^/ 0.056^*^/ 0.033^**^	0.037^**^/ 0.020/ 0.031^**^/ 0.021^**^	0.029/ 0.031/ 0.047^*^/ 0.033^*^	0.004/ 0.028^*^/ 0.062^*^/ 0.044^*^	0.006/ 0.011/ 0.002^**^/ 0.001^**^		
	**WC**	0.051^**^/ 0.073^*^/ 0.026^**^/ 0.010^**^	0.151^**^/ 0.229^**^/ 0.114^*^/ 0.064^*^	0.141^**^/ 0.242^**^/ 0.114^*^/ 0.065^*^	0.098^**^/ 0.118^**^/ 0.132/ 0.076^*^	0.140^**^/ 0.163^**^/ 0.105^*^/ 0.055^*^	0.031^**^/ 0.236^**^/ 0.097^*^/ 0.052^*^	
	SK	0.000/ 0.032 / -0.003^**^/ -0.002^**^	-0.010/ -0.008 / -0.001^**^/ 0.000^**^	0.008/ -0.005/ 0.003^**^/ 0.002^**^	0.005/ 0.008/ 0.026^**^/ 0.018^**^	0.021/ 0.047^*^/ 0.029^*^/ 0.018^**^	0.016/ 0.025/ 0.025^**^/ 0.016^**^	0.077^**^/ 0.127^**^/ 0.036^**^/ 0.017^**^

From top line, the values in Table indicate the *K*_ST_ and *F*_ST_, Hedrick's *G*'_ST_ and Jost's *D* statistics. Bold italic shows values which are statistically significant for both of *K*_ST_ and *F*_ST_, with positive values of *G*'_ST_ and *D*. Underline represents statistical significant *K*_ST_ and *F*_ST_ with more than 0.05 of *G*'_ST_ and *D*. Bold characters indicate conservation and management units which have more than 10 samples. Chokai Sanchi, Southern Ouu, Southern Alps and Kii Hanto units are excluded due to small number of samples. ^*^: *P* < 0.05; ^**^: *P* < 0.01 (permutation test, 1,000 replicates).

## Discussion

### Estimations of trans-species polymorphism in the *DQB* variants of the Japanese black bear

In this study, we detected 44 *DQB* variants in 185 Japanese black bears. These 44 nucleotide variants translated to give 31 allotypes and one putative, and the 31 allotypes were used to construct an ML tree to examine relationships among MHC class II sequences of ursine species (Figure 5). The tree showed that the DQB sequences of the Japanese black bear formed a monophyletic group with those of the giant panda. In our previous study, we reported that black bear DQB sequences cluster with DQB sequences of various mammalian species [39]. These results suggest that all MHC sequences of black bears are likely to have been derived from the *DQB* gene.

The ML tree suggested a pattern of DRB trans-species evolution among ursine species, in agreement with the Goda et al. [36] (Figure 5). The estimated divergence time of black bear *DQB* variants was approximately 11 ± 4.6 MYA. Previous studies have estimated that a rapid radiation of the genus *Ursus* has occurred at around 5 to 3 MYA [53,54]. Therefore, even if our estimate overestimated divergence time because of the limited number of synonymous sites, it is possible that the *DQB* allelic lineage of the Japanese black bear was shared with other species in the genus *Ursus*. In fact, Urth-DQB*0101 has been detected in the Asiatic black bear in Russia (YY, unpublished observation), although the PCR amplification indicating this was performed with the primer pair used in our previous study [39]. Fossil records suggest that the ancestral Japanese black bear migrated to the Japanese archipelago during the Middle Pleistocene (0.5 MYA - 0.3 MYA) [55]. Therefore, in the Asiatic black bear, including the Japanese black bear, the Urth-DQB*01 lineage may have been maintained for at least 0.3 million years (MY).

If sequences of other ursine species are obtained in the future, it will be important to carefully distinguish orthologous genes from paralogous ones. It is possible that variants of paralogous genes can aberrantly reflect the trans-specific pattern of alleles in orthologous genes.

### Selective pressure on the *DQB* gene of the Japanese black bear

To estimate whether balancing selection have acted on the *DQB* gene of black bears, we calculated *d*_N_/*d*_S_ (or *ω*) ratios by using three methods: the modified Nei–Gojobori model, ML, and Bayesian inferences. All three methods indicated that the *d*_N_/*d*_S_ values were significantly higher than unity, supporting the influence of balancing selection on the *DQB* locus of the Japanese black bear. According to the modified Nei–Gojobori method, the *d*_N_/*d*_S_ value in the PBR was 3.8. This value is relatively higher than those of some other mammalian species (Table 2). The values of the giant panda *DQB* (1.3), the Namibian cheetah *DRB* (2.0), and the European mink *DRB* (1.8) are all lower than that of the black bear. In addition, the number of MHC variants in the black bear is considerably higher than that of the three species even though the variants of the Namibian cheetah may be derived from multiple loci (Table 2). Since these mammalian species are all designated as an endangered species in the International Union for Conservation of Nature (IUCN) Red List [56], the effective population sizes of these species are expected to be quite small. In theory, the effect of selective pressure decreases with diminishing the effective population size, due to a larger effect of random genetic drift [57]. Thus, since the census population size of the Japanese black bear is relatively large, (more than 10,000 individuals; the exact number of individuals is still unclear.), the black bear may show the higher *d*_N_/*d*_S_ value, compared with other endangered species.

### Peptide-binding repertoires of the DQB molecules in the Japanese black bear

The amino acids in the PBRs for the Japanese black bear *DQB*s can be divided into 15 different types (Figure 4). Theoretically, the average number of nonsynonymous changes within the PBRs should be equal to the number of functionally different alleles that segregate in a population [58]. These groups might correspond to so-called “super-types” [59-61]. A super-type is a group of alleles with a similar repertoire of peptide binding. The mean number of nonsynonymous substitution in the PBRs (*K*_B_) of the black bear *DQB* was 10.3, and this translated to an expected number of allelic lineages of approximately ten. This is slightly inconsistent with the number of different types in the PBRs in Figure 4. This suggests that some PBRs may be functionally similar (i.e., a same super-type). Alternatively, since the length of *DQB* sequences is limited to exon 2 only, the resolution of the *K*_B_ estimate may be lower.

### Effects of demographic events on the Japanese black bear population

We compared results obtained from analyses of the mtDNA [8] and *DQB* genetic markers to estimate demographic events that have occurred in Japanese black bear populations. MHC alleles are maintained for long periods by balancing selection [62-64], while the coalescence times of mtDNA are short because of its maternal inheritance mode and ploidy level. Due to this characteristic, mtDNA has a higher susceptibility to the effect of recent demographic factors. Therefore, the time scales in evolutionary history represented by these two types of genetic markers are different.

The extent of sharing DQB allotypes among Japanese black bear management units was higher than that of mtDNA haplotypes (Figure 1). In particular, Urth-DQB*01 (Urth-DQB*0101) was frequently found throughout Japan. This observation could reflect the difference in coalescence time between the two systems and a time-dependent difference in the degree of population fragmentation. Alternatively, if the same dominant pathogen affects different populations (i.e., a similar selective pressure), a DQB allotype that confers resistance to this pathogen is expected to be shared by different populations. On the other hand, the management unit-specific allotypes were found from most of the units. This suggests that rare allotypes also have been maintained in the Japanese black bear population, possibly due to negative frequency-dependent selection [64].

Values of observed heterozygosity (*H*_o_) were lower than the *H*_e_ for all six units. Some management units showed a significant departure from Hardy-Weinberg equilibrium (HWE) (Table 4). The excess of *H*_e _appeared not to be associated with the quality of DNA samples as there was no correlation between the number of homozygotes and the quality of DNA samples (*P* = 0.90). In addition, to examine the possibility of null alleles, we determined nucleotide sequences of the primer target region in 96 samples (YY, unpublished data). While all individuals showed only one degenerated site in the reverse primer-annealing region, 10 individuals possessed seven degenerated sites in the forward primer region. In the forward primer region of the 10 individuals, there were two sets of nucleotide sequences that differed by less than 7 bp. The seven degenerated sites are derived from these two nucleotide sequences. However, the forward primer used in the present study (URDQBex2-F4) is designed to specifically anneal to only one particular sequence (see Methods) in the primer region. Therefore, the seven degenerated sites are unlikely to be the cause of null allele. This suggests that the excess of *H*_e _is not caused by technical problems. Since cross-locus amplification potentially decreases the possibility of the *H*_e _excess (i.e., it increases the *H*_o _values because of false heterozygotes), the *H*_e _excess suggests that our designed primer amplifies a single *DQB* locus. Although we cannot completely exclude the possibility of null alleles, the *H*_e _excess is more likely caused by demographic events such as non-random mating and/or population subdivision in a local population.

In our previous study [8], *F*_ST _values for mtDNA haplotypes among the management unit pairs in western Japan were larger than those among the pairs in eastern Japan. Since mtDNA haplotype distributions reflect recent demographic events, the large *F*_ST _in western Japan indicates a more infrequent gene flow within western Japanese populations. The level of genetic differentiation at *DQB* among the units in western Japan was also high. This increase may be due to non-random mating and genetic drift in western Japan in recent history. Non-random mating and genetic drift may be caused by fragmentation and isolation of habitat.

The genealogical structure of MHC alleles under strong symmetric balancing selection is similar to that of neutral genes, with the exception of a difference in time scales [65]. To estimate the effect of population subdivision in the Japanese black bear in the distant past, we calculated the ratio of the largest genetic distance (TMRCA, *d*_TMRCA_) to the mean distance (*d*_S_ or *d*) in bear *DQB* variants. At neutral genes, *d*_TMRCA _is expected to be twice the value of *d*[66]. The ratio of *d*_TMRCA _to *d*_S _in the *DQB* variants was approximately 3 (*d*_TMRCA_ = 0.078 ± 0.032, *d*_S_ = 0.028 ± 0.013), suggesting an effect of population fragmentation. Thus, the Japanese black bear population might have been isolated and fragmented not only in the recent past but also in the relatively distant past.

### Genetic degradation of black bears in the WC unit

For the WC unit in western Japan, values of genetic diversity, for not only the MHC *DQB*s (*H*_e_ = 0.33, *π* = 0.005) but also mtDNA (*h* = 0.56, *π* = 0.005; [8]), were significantly lower than those for the other management units (Table 4). In addition, both *F*_ST _and *K*_ST _values calculated between the WC unit and other units were significantly high, and also Hedrick’s *G’*_ST _and Jost’*D* tended to be higher than those among the other units. This suggests that genetic drift and/or non-random mating also influence MHC diversity in the WC unit, and it implies that black bears in the WC unit might be gradually diminishing in their potential to adapt to environmental adversity, such as the presence of various pathogens. Thus, even if bears in the WC unit currently have resistance against infectious disease, we need to design a conservation plan to maintain MHC diversity in the bears in this unit. The habitat area of the Eastern Chugoku (SK), Kii Hanto (KH), and Shikoku (SK) units in western Japan are smaller than that of the WC unit. Since previous studies that examined genetic diversities of black bears by using mtDNA and microsatellite markers have suggested a lower level of genetic diversities in the populations of western Japan [6,8], MHC variation in these management units might be lower than that in the WC unit. Therefore, it is essential to investigate MHC variation in these units further by using an increased number of samples.

### The effects of natural selection and genetic drift on patterns of MHC variation

We observed significant *H*_e _excess and genetic differentiation among the management units due to population fragmentation. Ohnishi et al. [6] examined genetic diversity of Japanese black bears by using 10 microsatellite makers. In the previous study, the deviation of *H*_o _from *H*_e _was not statistically significant in all examined local populations by using the HWE test. However, microsatellite markers that were not used in the study of Ohnishi et al. [6] showed such a deviation in the Eastern Chugoku (EC) and WC units in western Japan [4]. In addition, the genetic diversities within these units were similar to that of the isolated population of the brown bear [6]. Sutton et al. [67] proposed that negative frequency-dependent selection may be predominant in pre-bottlenecked populations and that greater loss of MHC diversity compared to neutral genetic diversity is accelerated because of uneven allele distributions. Such skewed MHC allele frequencies can occur under negative frequency-dependent selection or joint action of this selection [67,68]. Indeed, numerous MHC rare variants of the Japanese black bear were observed in the present study. Therefore, Japanese black bears might have lost some *DQB* rare variants due to a bottleneck event in their evolutionary history.

### Comparison of two criteria for MHC genotyping

Due to the possibility of PCR artifacts, criteria for MHC genotyping on the basis of partial sequences (e.g., exon 2 only) remain a matter of debate. For instance, previous studies have identified MHC variants according to more conservative criteria: If a rare sequence is detected in one individual alone (heterozygote) and the sequence differs by less than 3 bp from a known sequence of the same PCR product, the sequence is considered to be an artifact introduced by PCR error [69,70]. Although we attempted to carefully identify *DQB* variants, the methods might not be definitive. Therefore, we compared the results of analyses between the two criteria. According to our criteria, we detected 44 variants from 185 bears. Application of more conservative criteria reduced the number to 23 variants and to 179 bear samples. Nevertheless, results of analyses such as estimates of *d*_N_/*d*_S _ratio, genetic differentiation among units and deviation from the HWE did not largely change even though the number of variants was different between two criteria. This suggests that our conclusion is not affected by the differences between criteria.

### Future studies

In this study, we detected a maximum of 44 variants including one putative (23 at a minimum). However, there is a possibility that some of these variants are pseudogenized (i.e., contain frameshift or nonsense mutations in other exons or promoter regions), although we confirmed that the nucleotide sequence of exon 2 in a near full-length cDNA sequence was identical to that of the Urth-DQB*01 [39]. Since a majority of our samples was collected from tooth, the size of amplified DNA fragments was limited to the 270 bp of exon 2. In addition, the sample size of our study was not sufficient to investigate MHC diversity in each management unit. To evaluate our results precisely, further analysis based on the sequences of the entire coding region of *DQB* should be performed by using more samples.

We designed two pairs of primers to amplify the bear *DQB* exon 2. When one of these pairs (URDQBex2-F1 and -R1) was used, three different sequences were detected from an individual by TA cloning, suggesting that at least two *DQB* loci were amplified. Wan et al. [31] have reported that the giant panda genome has three *DQB* loci, two functional and one non-functional. Kuduk et al. [37] have also confirmed expression of two *DQB* genes in the brown bear. The PCR results of our study suggest that the Japanese black bear may also possess at least two *DQB* loci in the genome (See Methods). It will be desirable to determine the genomic sequence across the wider MHC region in order to assure the specificity of each MHC locus in the bear genome.

## Conclusions

In the present study, we detected 44 MHC variants from 185 Japanese black bears. The phylogenetic analysis suggests that their sequences are derived from the *DQB* loci. The value of *d*_N_/*d*_S _ratio indicates that the bear *DQB* variants have been maintained by balancing selection. The estimation of TMRCA among their variants suggests that the *DQB* lineages arose before the origin of genus *Ursus*. At least, the Urth-DQB*0101 variant has been shared between Japanese and continental black bears for 0.3 MY (before the migration of Asiatic black bear from the Asian continent into Japanese archipelago). However, *DQB* genotyping for the Japanese black bear showed considerably lower *H*_o _values than *H*_e _values in each local population. One may hypothesize that skewed allelic distribution due to negative frequency-dependent selection and the bottleneck or population fragmentation in each management unit might accelerate loss of *DQB* rare variants. Therefore, the results of our study imply that MHC variation in Japanese black bears might have declined due to demographic events such as habitat fragmentation and isolation at the population or species level. Nevertheless, *DQB* genetic diversity in Japanese black bears appears to be relatively high compared to some other endangered mammalian species. This result suggests that for the present the Japanese black bears appear to retain more potential resistance against pathogens than other endangered mammalian species. To prevent further decline of potential resistance against pathogens, a conservation policy for the Japanese black bear should be designed to maintain MHC rare variants in each of management units. To achieve this objective, it is important to stop further fragmentation and isolation of bear habitats.

## Methods

### Collection of samples from Japanese black bears

In this study, we collected 307 Japanese black bear samples throughout the Japanese archipelago. The samples were collected from individuals that were hunted or captured for pest control. Of these samples, 185 were used for MHC genotyping. These 185 samples were obtained from 12 conservation and management units designated by Yoneda [42] (Figure 1) between 1970 and 2006. Local populations of the black bears in Japan are divided into 19 conservation and management units, taking into account the prevention of bear movement, such as rivers, railways and so on. The division of local populations in our study followed the divisions of the bear management units. Five samples were obtained from the Chokai Sanchi (CS) unit; 21 from the Gassan-Asahi Iide (GAI) unit; one from the Southern Ouu (SO) unit; 13 from the Echigo-Mikuni (EM) unit; one from the Southern Alps (SA) unit; 12 from the North-Central Alps (NCA) unit; 36 from the Hakusan-Okumino (HO) unit; 23 from the Northern Kinki (NK) unit; 8 from the Eastern Chugoku (EC) unit; 53 from the Western Chugoku (WC) unit; 3 from the Kii Hanto (KH) unit; and 9 from the Shikoku (SK) unit (Figure 1). We obtained the following types of samples: muscle and liver tissue, 42 samples; blood, 10; hair, 12; oral mucous membrane, 4; and tooth or mandibula bone, 117. Based on the mtDNA analysis (see above), there were three genetically distinct groups of Japanese black bears. However, the sample sizes of the management units in the Shikoku/Kii-hanto populations were quite small in this study (three samples in the KH unit and nine in the SK unit). Therefore, for the purpose of discussion of population structure, we combined the Shikoku/Kii-hanto population with the western Japanese population, as is the case with our previous study [8].

### Experimental procedures for MHC genotyping

DNA extraction was performed using the procedure described by our previous study [8]. To amplify the entire exon 2 of the *DQB* in the Japanese black bear, we initially used a single primer set URDQBex2-F1 and URDQBex2-R1 [39]. However, when this primer set was used, three variants per individual were detected from several individuals (i.e., at least two loci were amplified). When nucleotide sequences of the three variants were compared, two of three variants showed nearly identical sequences. Therefore, for amplification of a single *DQB* locus, a pair of internal primers for introns 1 and 2, URDQBex2-F4 (5′-GAGGCTGTGGGTTGGCCTGAGGCGCGGA-3′) and URDQBex2-R4 (5′-CCGCGGGGCGCGAACGGCCTGGCTCA-3′), was designed to specifically amplify the sequences in intron 1 region of their two similar variants. The PCR amplification, identification of heterozygous or homozygous individuals by TA cloning, and sequencing were performed as described by Yasukochi et al. [39]. Murray et al. [71] showed that the probability that both of two variant sequences in a heterozygote are not detected by sequencing five clones (assuming that every variant is equally amplified at an equal frequency) is very low (*P* = 0.06). In our study, all final PCR products (188) were cloned, and 8 to 22 clones per individual were sequenced to decrease the possibility of false homozygotes caused by amplification bias. In addition, to confirm sequencing reliability, we compared the result of direct-sequencing at the polymorphic sites in uncloned PCR product with cloned sequences (see Additional File 2: Figure S2). Since such a direct-sequencing are derived from the PCR product amplified from an original gDNA, it decreases the possibility of error incurred during cloning or PCR of the plasmid insert after cloning. If the frequencies of two variants detected from a heterozygote were extremely different and a double peak corresponding to the correct nucleotides was not observed after direct sequencing, that individual was excluded from our analyses (see Additional File 2: Figure S2). In addition, we used high fidelity polymerase during all PCRs (Ex Taq Hot Start Version TaKaRa Bio Inc.).

### Phylogenetic relationships and population differentiation

The nucleotide sequences were aligned and translated into amino acids by using MEGA v5.0 [72]. The NJ and ML trees were constructed on the basis of Jones-Taylor-Thornton (JTT) model with gamma distribution [73]. The NJ tree construction and estimation of the best fit substitution model were performed by using the same software. The ML tree was estimated by using the PhyML 3.0 [74]. Bootstrap analysis was performed (1,000 replications in the NJ tree and 500 replications in the ML tree). A network of MHC variants was constructed with the Neighbor-Net method [75] by using Splits-tree4 ver. 4.11.3 [76]. A Bayesian phylogenetic analysis was conducted with MrBayes v3.2.1 [77]. The analysis was implemented considering 11,500,000 generations and tree sampling every 100 generations. The first 28,750 trees were discarded as burn-in. The genetic distance was estimated by using the JTT model. The Bayesian and ML trees were visualized with FigTree version 1.3.1 (http://tree.bio.ed.ac.uk/software/figtree/, [78]). The expected heterozygosity (*H*_e_) for each management unit and Wright’s *F*-statistics (*F*_ST_) between the pairs of management units were calculated using Arlequin v3.11 [79]. In addition, we calculated *K*_ST_[80] that, like *F*_ST_, is an index of genetic differentiation, using the DnaSP 5.10 [81]. *F*_ST _is based on frequencies at polymorphic sites, while *K*_ST _uses the average number of differences between sequences. The *F*_ST _and *K*_ST _are possibly misleading regarding the differentiation when heterozygosity is high. Thus, we also calculated Hedrick’s *G*’_ST_[82] and Jost’s *D*[83] using the SMOGD 1.2.5 [84].

### Measure of genetic diversity and distance among *DQB* variants

The extent of amino acid variability was evaluated using a Wu–Kabat plot [85]. The mean numbers of nonsynonymous substitutions per nonsynonymous site (*d*_N_) and that of synonymous substitutions per synonymous site (*d*_S_) were calculated for excluding identical sequences of *DQB* variants. We estimated nucleotide diversity (*π*_S_) among all the sequences in each management unit or across the entire population. The divergence time of the Japanese black bear *DQB* variants was estimated using synonymous substitutions alone. We assumed that the maximum genetic distance at synonymous sites roughly corresponded to the TMRCA of *DQB*s. These genetic distance parameters (*π*_S_, *d*_N_, and *d*_S_) were calculated using the modified Nei–Gojobori method [47] with Jukes–Cantor correction [86] by using MEGA v5.0. The transition/transversion bias in the calculation was assumed as *R* = 1.58 by the ML method. Standard errors were calculated from 1,000 bootstrapping replicates.

### Estimation of natural selection for *DQB* variants

Ratios of *d*_N_/*d*_S_ were calculated for each of the putative PBRs, non-PBRs, and for the overall exon 2 region. The extent of selective pressure at the amino acid level was measured by the ratio of nonsynonymous to synonymous rates (*ω* = *d*_N_/*d*_S_). We estimated *ω* ratios with the maximum likelihood method by using CODEML in the PAML program version 4.2b [50]. Several models that differed in their hypotheses concerning the statistical distributions of the *ω* ratio [48,49] were considered in this study. Nested models can be compared in pairs by using a likelihood-ratio test [87]. A Bayesian approach (Bayes empirical Bayes) in CODEML is usually used to predict codons that were potentially under positive selection or balancing selection in models M2a and M8 [49]. Although it is difficult to distinguish between positive selection and balancing selection, we use this Bayesian approach to estimate sites under balancing selection because MHC genes are thought to be affected by balancing selection rather than positive selection [65,88]. The ML method has the potential to overestimate *ω* values because of the existence of recombination [70]. Therefore, we estimated the *ω* value by using a Bayesian method with OmegaMap [51], which enabled us to infer balancing selection in the presence of recombination. The average number of nonsynonymous substitutions in the PBRs (*K*_B_) was also estimated using method II, as described by Satta et al. [89].

### Test for reciprocal recombination or gene conversion

The GENECONV program was used with default settings. The global test for reciprocal recombination or gene conversion events was performed with 10,000 permutations of the sequence alignment to assess significance. Moreover, we examined the possibility of recombination by using the method described by Satta [44]. In the method, it is assumed that the number of substitutions in a particular region to the entire region is binomially distributed. We divided exon 2 into two regions, *β* sheet and *α* helix, for the examination of recombination. The RDP4 software was also used for detecting a possible recombination [45].

## Abbreviations

MHC: Major histocompatibility complex; PBR: Peptide-binding residue; NJ: Neighbor-joining; TMRCA: Time to most recent common ancestor; MY: Million years; MYA: Million years ago; *H*_e_: Expected heterozygosity; *H*_O_: Observed heterozygosity; ML: Maximum likelihood; JTT: Jones-Taylor-Thornton; HWE: Hardy-Weinberg equilibrium; CS: Chokai Sanchi; GAI: Gassan-Asahi Iide; SO: Southern Ouu; EM: Echigo-Mikuni; SA: Southern Alps; NCA: North-Central Alps; HO: Hakusan-Okumino; NK: Northern Kinki; EC: Eastern Chugoku; WC: Western Chugoku; KH: Kii Hanto; SK: Shikoku.

## Competing interests

The authors declare that they have no competing interests.

## Authors' contributions

YY carried out the experiments and data analysis, and wrote the draft of manuscript. TK and MY participated in sample collection. HK and YS were the primary supervisors. All authors have read and approved the final manuscript.

## Supplementary Material

Additional file 1 **The NJ and Bayesian trees of ursine *DQB *and *DRB *genes.** The NJ and Bayesian trees were constructed based on amino acid sequences of partial exon 2 region of ursine *DQB* or *DRB* genes. The JTT model with gamma distribution is used for the NJ and Bayesian trees, respectively. Numbers in parentheses are Genbank accession numbers. Only bootstrap values over 50% and posterior probabilities over 80% are shown in the NJ and Bayesian trees, respectively. Click here for file

Additional file 2 **The comparison of nucleotides at the polymorphic sites between uncloned and cloned PCR products.** (A) An example that an individual is identified as a heterozygote. The upper figure is the alignment of nucleotide sequences of uncloned and cloned PCR products. The lower figure is the result of direct-sequencing for uncloned PCR product. (B) An example that an individual is not identified as a heterozygote. Such an individual is excluded from analyses of the present study. Click here for file

## References

[B1] Japan Wildlife Research Center Report on population status of Japanese bears in 1998 1999 Japan Wildlife Research Center in Japanese

[B2] Ohnishi N Yasukochi Y The origin of the last Asian black bear in Kyushu Island Mamm Sci 2010 50 177 180 in Japanese, with English summary

[B3] Kurihara T Records of recent bear witnesses in Kyushu Island, Japan Mamm Sci 2010 50 187 193 in Japanese, with English summary

[B4] Saitoh T Ishibashi Y Kanamori H Kitahara E Genetic status of fragmented populations of the Asian black bear Ursus thibetanus in western Japan Popul Ecol 2001 43 221 227 10.1007/s10144-001-8186-4

[B5] Ishibashi Y Saitoh T Phylogenetic relationships among fragmented Asian black bear (Ursus thibetanus) populations in western Japan Conservat Genet 2004 5 311 323

[B6] Ohnishi N Saitoh T Ishibashi Y Oi T Low genetic diversities in isolated populations of the Asian black bear (Ursus thibetanus) in Japan, in comparison with large stable populations Conservat Genet 2007 8 1331 1337 10.1007/s10592-006-9281-z

[B7] Ohnishi N Uno R Ishibashi Y Tamate HB Oi T The influence of climatic oscillations during the Quaternary Era on the genetic structure of Asian black bears in Japan Heredity 2009 102 579 589 10.1038/hdy.2009.28 19319151

[B8] Yasukochi Y Nishida S Han S-H Kurosaki T Yoneda M Koike H Genetic structure of the Asiatic black bear in Japan using mitochondrial DNA analysis J Hered 2009 100 297 308 10.1093/jhered/esn097 18984857

[B9] Ohnishi N Yuasa T Morimitsu Y Oi T Mass-intrusion-induced temporary shift in the genetic structure of an Asian black bear population Mamm Stud 2011 36 67 71 10.3106/041.036.0204

[B10] Yoneda M Mano T The present situation and issues for estimating population size and monitoring population trends of bears in Japan Mamm Sci 2011 51 79 95 in Japanese, with English summary

[B11] Yamamoto1 T Oka T Ohnishi N Tanaka H Takatsuto N Okumura Y Genetic Characterization of Northernmost Isolated Population of Asian Black Bear (Ursus thibetanus) in Japan Mamm Stud 2012 37 85 91 10.3106/041.037.0209

[B12] Uno R Kondo M Yuasa T Yamauchi K Tsuruga H Tamate HB Yoneda M Assessment of genotyping accuracy in a non-invasive DNA-based population survey of Asiatic black bears (Ursus thibetanus): lessons from a large-scale pilot study in Iwate prefecture, northern Japan Popul Ecol 2012 54 509 519 10.1007/s10144-012-0328-3

[B13] Ministry of the Environment Threatened Wildlife of Japan—Red Data Book Mammalia 2002 2 Japan Wildlife Research Center 1 in Japanese

[B14] Klein J Takahata N The major histocompatibility complex and the quest for origins Immunol Rev 1990 113 5 25 10.1111/j.1600-065X.1990.tb00034.x 2180811

[B15] Man B Complete sequence and gene map of a human major histocompatibility complex. The MHC sequencing consortium Nature 1999 401 921 923 10.1038/44853 10553908

[B16] Delisle I Strobeck C A phylogeny of the Caniformia (order Carnivora) based on 12 complete protein-coding mitochondrial genes Mol Phylogenet Evol 2005 37 192 201 10.1016/j.ympev.2005.04.025 15964215

[B17] Yu L Luan PT Jin W Ryder OA Chemnick LG Davis HA Zhang YP Phylogenetic utility of nuclear introns in interfamilial relationships of Caniformia (order Carnivora) Syst Biol 2011 60 175 187 10.1093/sysbio/syq090 21252386

[B18] Hoelzel AR Stephens JC O’Brien SJ Molecular genetic diversity and evolution at the MHC DQB locus in four species of pinnipeds Mol Biol Evol 1999 16 611 618 10.1093/oxfordjournals.molbev.a026143 10335654

[B19] Lento GM Baker CS David V Yuhki N Gales NJ O’Brien SJ Automated single-strand conformation polymorphism reveals low diversity of a Major Histocompatibility Complex Class II gene in the threatened New Zealand sea lion Mol Ecol Notes 2003 3 346 349 10.1046/j.1471-8286.2003.00445.x

[B20] Weber DS Stewart BS Schienman J Lehman N Major histocompatibility complex variation at three class II loci in the northern elephant seal Mol Ecol 2004 13 711 718 10.1111/j.1365-294X.2004.02095.x 14871373

[B21] Froeschke G Sommer S MHC class II DRB variability and parasite load in the striped mouse (Rhabdomys pumilio) in the Southern Kalahari Mol Biol Evol 2005 22 1254 1259 10.1093/molbev/msi112 15703235

[B22] Smith S Belov K Hughes J MHC screening for marsupial conservation: extremely low levels of class II diversity indicate population vulnerability for an endangered Australian marsupial Conservat Genet 2009 11 269 278

[B23] Bowen L Aldridge BM Gulland F Woo J Van Bonn W DeLong R Stott JL Johnson ML Molecular characterization of expressed DQA and DQB genes in the California sea lion (Zalophus californianus) Immunogenetics 2002 54 332 347 10.1007/s00251-002-0472-6 12185537

[B24] Bowen L Aldridge BM Gulland F Van Bonn W DeLong R Melin S Lowenstine LJ Stott JL Johnson ML Class II multiformity generated by variable MHC-DRB region configurations in the California sea lion (Zalophus californianus) Immunogenetics 2004 56 12 27 10.1007/s00251-004-0655-4 14997355

[B25] Siddle HV Marzec J Cheng Y Jones M Belov K MHC gene copy number variation in Tasmanian devils: implications for the spread of a contagious cancer Proc Biol Sci 2010 277 2001 2006 10.1098/rspb.2009.2362 20219742 PMC2880097

[B26] Lane A Cheng Y Wright B Hamede R Levan L Jones M Ujvari B Belov K New insights into the role of MHC diversity in devil facial tumour disease PLoS One 2012 7 e36955 10.1371/journal.pone.0036955 22701561 PMC3368896

[B27] Mainguy J Worley K Côté SD Coltman DW Low MHC DRB class II diversity in the mountain goat: past bottlenecks and possible role of pathogens and parasites Conservat Genet 2007 8 885 891 10.1007/s10592-006-9243-5

[B28] Zeng CJ Yu JQ Pan HJ Wan QH Fang SG Assignment of the giant panda MHC class II gene cluster to chromosome 9q by fluorescence in situ hybridization Cytogene Genome Res 2005 109 533 10.1159/000084222 15906477

[B29] Zeng CJ Pan HJ Gong SB Yu JQ Wan QH Fang SG Giant panda BAC library construction and assembly of a 650-kb contig spanning major histocompatibility complex class II region BMC Genom 2007 8 315 10.1186/1471-2164-8-315 PMC2018726 17825108

[B30] Wan QH Zhu L Wu H Fang SG Major histocompatibility complex class II variation in the giant panda (Ailuropoda melanoleuca) Mol Ecol 2006 15 2441 2450 10.1111/j.1365-294X.2006.02966.x 16842418

[B31] Wan QH Zeng CJ Ni XW Pan HJ Fang SG Giant panda genomic data provide insight into the birth-and-death process of mammalian major histocompatibility complex class II genes PLoS One 2009 4 11 10.1371/journal.pone.0004147 PMC2613555 19127303

[B32] Zhu L Ruan XD Ge YF Wan QH Fang SG Low major histocompatibility complex class II DQA diversity in the Giant Panda (Ailuropoda melanoleuca) BMC Genet 2007 8 29 17555583 10.1186/1471-2156-8-29 PMC1904234

[B33] Pan HJ Wan QH Fang SG Molecular characterization of major histocompatibility complex class I genes from the giant panda (Ailuropoda melanoleuca) Immunogenetics 2008 60 185 193 10.1007/s00251-008-0281-7 18292994

[B34] Chen YY Zhang YY Zhang HM Ge YF Wan QH Fang SG Natural selection coupled with intragenic recombination shapes diversity patterns in the major histocompatibility complex class II genes of the giant panda J Exp Zool B Mol Dev Evol 2010 314 208 223 19950128 10.1002/jez.b.21327

[B35] Goda N Mano T Masuda R Genetic diversity of the MHC class-II DQA gene in brown bears (Ursus arctos) on Hokkaido, Northern Japan Zoolog Sci 2009 26 530 535 10.2108/zsj.26.530 19719404

[B36] Goda N Mano T Kosintsev P Vorobiev A Masuda R Allelic diversity of the MHC class II DRB genes in brown bears (Ursus arctos) and a comparison of DRB sequences within the family Ursidae Tissue Antigens 2010 76 404 410 10.1111/j.1399-0039.2010.01528.x 20630039

[B37] Kuduk K Babik W Bojarska K Sliwinska EB Kindberg J Taberlet P Swenson JE Radwan J Evolution of major histocompatibility complex class I and class II genes in the brown bear BMC Evol Biol 2012 12 197 10.1186/1471-2148-12-197 23031405 PMC3508869

[B38] Aguilar A Roemer G Debenham S Binns M Garcelon D Wayne RK High MHC diversity maintained by balancing selection in an otherwise genetically monomorphic mammal Proc Nat Acad Sci USA 2004 101 3490 3494 10.1073/pnas.0306582101 14990802 PMC373489

[B39] Yasukochi Y Kurosaki T Yoneda M Koike H Identification of the expressed MHC class II DQB gene of the Asiatic black bear, Ursus thibetanus, in Japan Gene Genet Syst 2010 85 147 155 10.1266/ggs.85.147 20558901

[B40] Brown JH Jardetzky TS Gorga JC Stern LJ Urban RG Strominger JL Wiley DC Three-dimensional structure of the human class II histocompatibility antigen HLA-DR1 Nature 1993 364 33 39 10.1038/364033a0 8316295

[B41] Stern LJ Wiley DC Antigenic peptide binding by class I and class II histocompatibility proteins Structure 1994 2 245 251 10.1016/S0969-2126(00)00026-5 8087551

[B42] Yoneda M The local population division, conservation and management for the Asiatic black bear Landscape Stud 2001 64 314 317 in Japanese

[B43] Sawyer S Statistical tests for detecting gene conversion Mol Biol Evol 1989 6 526 538 2677599 10.1093/oxfordjournals.molbev.a040567

[B44] Satta Y Takahata N Balancing selection at HLA loci The Proceedings of the 17th Taniguchi Symposium 1992 Science Society Press, Tokyo: Japan 111 131

[B45] Martin DP Lemey P Lott M Moulton V Posada D Lefeuvre P RDP3: a flexible and fast computer program for analyzing recombination Bioinformatics (Oxford, England) 2010 26 2462 2463 10.1093/bioinformatics/btq467 PMC2944210 20798170

[B46] Saitou N Nei M The neighbor-joining method: a new method for reconstructing phylogenetic trees Mol Biol Evol 1987 4 406 425 3447015 10.1093/oxfordjournals.molbev.a040454

[B47] Zhang J Rosenberg HF Nei M Positive Darwinian selection after gene duplication in primate ribonuclease genes Proc Nat Acad Sci USA 1998 95 3708 3713 10.1073/pnas.95.7.3708 9520431 PMC19901

[B48] Yang Z Nielsen R Goldman N Pedersen AM Codon-substitution models for heterogeneous selection pressure at amino acid sites Genetics 2000 155 431 449 10790415 10.1093/genetics/155.1.431 PMC1461088

[B49] Yang Z Wong WSW Nielsen R Bayes empirical Bayes inference of amino acid sites under positive selection Mol Biol Evol 2005 22 1107 1118 10.1093/molbev/msi097 15689528

[B50] Yang Z PAML 4: phylogenetic analysis by maximum likelihood Mol Biol Evol 2007 24 1586 1591 10.1093/molbev/msm088 17483113

[B51] Wilson DJ McVean G Estimating diversifying selection and functional constraint in the presence of recombination Genetics 2006 172 1411 1425 16387887 10.1534/genetics.105.044917 PMC1456295

[B52] Li W Molecular Evolution 1997 Sunderland, Massachusetts: Sinauer Associates

[B53] Krause J Unger T Noçon A Malaspinas AS Kolokotronis SO Stiller M Soibelzon L Spriggs H Dear PH Briggs AW Bray SC O’Brien SJ Rabeder G Matheus P Cooper A Slatkin M Pääbo S Hofreiter M Mitochondrial genomes reveal an explosive radiation of extinct and extant bears near the Miocene-Pliocene boundary BMC Evol Biol 2008 8 220 10.1186/1471-2148-8-220 18662376 PMC2518930

[B54] Bon C Caudy N De Dieuleveult M Fosse P Philippe M Maksud F Beraud-Colomb É Bouzaid E Kefi R Laugier C Rousseau B Casane D Van Der Plicht J Elalouf JM Deciphering the complete mitochondrial genome and phylogeny of the extinct cave bear in the Paleolithic painted cave of Chauvet Proc Nat Acad Sci USA 2008 105 17447 17452 10.1073/pnas.0806143105 18955696 PMC2582265

[B55] Dobson M Kawamura Y Origin of the Japanese land mammal fauna: allocation of extant species to historically-based categories Quat Res 1998 37 385 395 10.4116/jaqua.37.385

[B56] IUCN Red List of Threatened Species; Version 2012.8 [www.iucnredlist.org]

[B57] Frankham R Ballou JD Briscoe DA Introduction to Conservation Genetics 2002 Cambridge: Cambridge University Press

[B58] Takahata N Satta Y Klein J Polymorphism and balancing selection at major histocompatibility complex loci Genetics 1992 130 925 938 1582567 10.1093/genetics/130.4.925 PMC1204941

[B59] Del Guercio MF Sidney J Hermanson G Perez C Grey HM Kubo RT Sette A Binding of a peptide antigen to multiple HLA alleles allows definition of an A2-like supertype J Immunol 1995 154 685 693 7529283

[B60] Sidney J Del Guercio MF Southwood S Engelhard VH Appella E Rammensee HG Falk K Rötzschke O Takiguchi M Kubo RT Several HLA alleles share overlapping peptide specificities J Immunol 1995 154 247 259 7527812

[B61] Greenbaum J Sidney J Chung J Brander C Peters B Sette A Functional classification of class II human leukocyte antigen (HLA) molecules reveals seven different supertypes and a surprising degree of repertoire sharing across supertypes Immunogenetics 2011 63 325 335 10.1007/s00251-011-0513-0 21305276 PMC3626422

[B62] Hughes AL Nei M Pattern of nucleotide substitution at major histocompatibility complex class I loci reveals overdominant selection Nature 1988 335 167 170 10.1038/335167a0 3412472

[B63] Hughes AL Nei M Nucleotide substitution at major histocompatibility complex class II loci: evidence for overdominant selection Proc Nat Acad Sci USA 1989 86 958 962 10.1073/pnas.86.3.958 2492668 PMC286598

[B64] Takahata N Nei M Allelic genealogy under overdominant and frequency-dependent selection and polymorphism of major histocompatibility complex loci Genetics 1990 124 967 978 2323559 10.1093/genetics/124.4.967 PMC1203987

[B65] Takahata N A simple genealogical structure of strongly balanced allelic lines and trans-species evolution of polymorphism Proc Nat Acad Sci USA 1990 87 2419 2423 10.1073/pnas.87.7.2419 2320564 PMC53700

[B66] Satta Y Takahata N, Clark A Balancing selection at HLA loci Mechanisms of molecular evolution 1993 Sunderland, Massachusetts: Sinauer Associates 129 149

[B67] Sutton JT Nakagawa S Robertson BC Jamieson IG Disentangling the roles of natural selection and genetic drift in shaping variation at MHC immunity genes Mol Ecol 2011 20 4408 20 10.1111/j.1365-294X.2011.05292.x 21981032

[B68] Ejsmond MJ Babik W Radwan J MHC allele frequency distributions under parasite-driven selection: A simulation model BMC Evol Biol 2010 10 332 10.1186/1471-2148-10-332 20979635 PMC2978226

[B69] Edwards SV Grahn M Potts WK Dynamics of Mhc evolution in birds and crocodilians: amplification of class II genes with degenerate primers Mol Ecol 1995 4 719 729 10.1111/j.1365-294X.1995.tb00272.x 8564010

[B70] Alcaide M Edwards SV Negro JJ Serrano D Tella JL Extensive polymorphism and geographical variation at a positively selected MHC class II B gene of the lesser kestrel (Falco naumanni) Mol Ecol 2008 17 2652 2665 10.1111/j.1365-294X.2008.03791.x 18489548

[B71] Murray BW Malik S White BN Sequence variation at the major histocompatibility complex locus DQ beta in beluga whales (Delphinapterus leucas) Mol Biol Evol 1995 12 582 593 7659014 10.1093/oxfordjournals.molbev.a040238

[B72] Tamura K Peterson D Peterson N Stecher G Nei M Kumar S MEGA5: molecular evolutionary genetics analysis using maximum likelihood, evolutionary distance, and maximum parsimony methods Mol Biol Evol 2011 28 2731 2739 10.1093/molbev/msr121 21546353 PMC3203626

[B73] Jones D Taylor W Thornton J The rapid generation of mutation data matrices from protein sequences Comput Appl Biosci 1992 8 275 282 1633570 10.1093/bioinformatics/8.3.275

[B74] Guindon S Dufayard J-F Lefort V Anisimova M Hordijk W Gascuel O New algorithms and methods to estimate maximum-likelihood phylogenies: assessing the performance of PhyML 3.0 Syst Biol 2010 59 307 321 10.1093/sysbio/syq010 20525638

[B75] Bryant D Moulton V Neighbor-net: an agglomerative method for the construction of phylogenetic networks Mol Biol Evol 2004 21 255 265 14660700 10.1093/molbev/msh018

[B76] Huson DH Bryant D Application of phylogenetic networks in evolutionary studies Mol Biol Evol 2006 23 254 267 16221896 10.1093/molbev/msj030

[B77] Ronquist F Huelsenbeck JP MrBayes 3: Bayesian phylogenetic inference under mixed models Bioinformatics 2003 19 1572 1574 10.1093/bioinformatics/btg180 12912839

[B78] Rambaut A Tree Figure Drawing Tool Version 1.3.1 2009

[B79] Excoffier L Laval G Schneider S Arlequin ver. 3.0: An integrated software package for population genetics data analysis Evol Bioinformatics Online 2005 1 47 50 PMC2658868 19325852

[B80] Hudson RR Boos DD Kaplan NL A statistical test for detecting geographic subdivision Mol Biol Evol 1992 9 138 151 1552836 10.1093/oxfordjournals.molbev.a040703

[B81] Librado P Rozas J DnaSP v5: a software for comprehensive analysis of DNA polymorphism data Bioinformatics 2009 25 1451 1452 10.1093/bioinformatics/btp187 19346325

[B82] Hedrick PW A standardized genetic differentiation measure Evolution 2005 59 1633 1638 16329237

[B83] Jost L GST and its relatives do not measure differentiation Mol Ecol 2008 17 4015 4026 10.1111/j.1365-294X.2008.03887.x 19238703

[B84] Crawford NG SMOGD: Software for the Measurement of Genetic Diversity Mol Ecol Resour 2010 10 556 557 10.1111/j.1755-0998.2009.02801.x 21565057

[B85] Wu TT Kabat EA An analysis of the sequences of the variable regions of Bence Jones proteins and myeloma light chains and their implications for antibody complementarity J Exp Med 1970 132 211 250 10.1084/jem.132.2.211 5508247 PMC2138737

[B86] Jukes T Cantor C Munro H Evolution of protein molecules Mammalian protein metabolism 1969 New York: Academic Press 21 132

[B87] Nielsen R Yang Z Likelihood models for detecting positively selected amino acid sites and applications to the HIV-1 envelope gene Genetics 1998 148 929 936 9539414 10.1093/genetics/148.3.929 PMC1460041

[B88] Sommer S The importance of immune gene variability (MHC) in evolutionary ecology and conservation Front Zool 2005 2 16 10.1186/1742-9994-2-16 16242022 PMC1282567

[B89] Satta Y O’hUigin C Takahata N Klein J Intensity of natural selection at the major histocompatibility complex loci Proc Nat Acad Sci USA 1994 91 7184 7188 10.1073/pnas.91.15.7184 8041766 PMC44363

[B90] Nishida S Tachi T Baba Y Hayashi K Kawanami N Koike H DNA analysis of the Asiatic black bear in the Tohoku region using hair-trap samples. Report on model project of networking maintenance for habitat of wildlife 2002 Wildlife Research Center, Tokyo: Japan (in Japanese)

[B91] Marsden CD Woodroffe R Mills MGL McNutt JW Creel S Groom R Emmanuel M Cleaveland S Kat P Rasmussen GS Ginsberg J Lines R André JM Begg C Wayne RK Mable BK Spatial and temporal patterns of neutral and adaptive genetic variation in the endangered African wild dog (Lycaon pictus) Mol Ecol 2012 21 1379 1393 10.1111/j.1365-294X.2012.05477.x 22320891

[B92] Castro-Prieto A Wachter B Sommer S Cheetah paradigm revisited: MHC diversity in the world’s largest free-ranging population Mol Biol Evol 2011 28 1455 1468 10.1093/molbev/msq330 21183613 PMC7187558

[B93] Becker L Nieberg C Jahreis K Peters E MHC class II variation in the endangered European mink Mustela lutreola (L. 1761)–consequences for species conservation Immunogenetics 2009 61 281 288 10.1007/s00251-009-0362-2 19263000

[B94] Niranjan SK Deb SM Kumar S Mitra A Sharma A Sakaram D Naskar S Sharma D Sharma SR Allelic diversity at MHC class II DQ loci in buffalo (Bubalus bubalis): evidence for duplication Vet Immunol Immunopathol 2010 138 206 212 10.1016/j.vetimm.2010.07.014 20724005

[B95] Sena L Schneider MPC Brenig BB Honeycutt RL Honeycutt D Womack JE Skow LC Polymorphism and gene organization of water buffalo MHC-DQB genes show homology to the BoLA DQB region Anim Genet 2011 42 378 385 10.1111/j.1365-2052.2010.02157.x 21749420

[B96] Diaz D Naegeli M Rodriguez R Nino-Vasquez JJ Moreno A Patarroyo ME Pluschke G Daubenberger CA Sequence and diversity of MHC DQA and DQB genes of the owl monkey Aotus nancymaae Immunogenetics 2000 51 528 537 10.1007/s002510000189 10912504

[B97] Meyer-Lucht Y Sommer S MHC diversity and the association to nematode parasitism in the yellow-necked mouse (Apodemus flavicollis) Mol Ecol 2005 14 2233 2243 10.1111/j.1365-294X.2005.02557.x 15910340

