# Supplementary figures and images for: MHC class II DQB diversity in the Japanese black bear, Ursus thibetanus japonicus
Source: BMC Evol Biol. 2012 Nov 29;12:230. doi: 10.1186/1471-2148-12-230 (PMC3575356; doi:10.1186/1471-2148-12-230)

# NJ tree

DQB clade

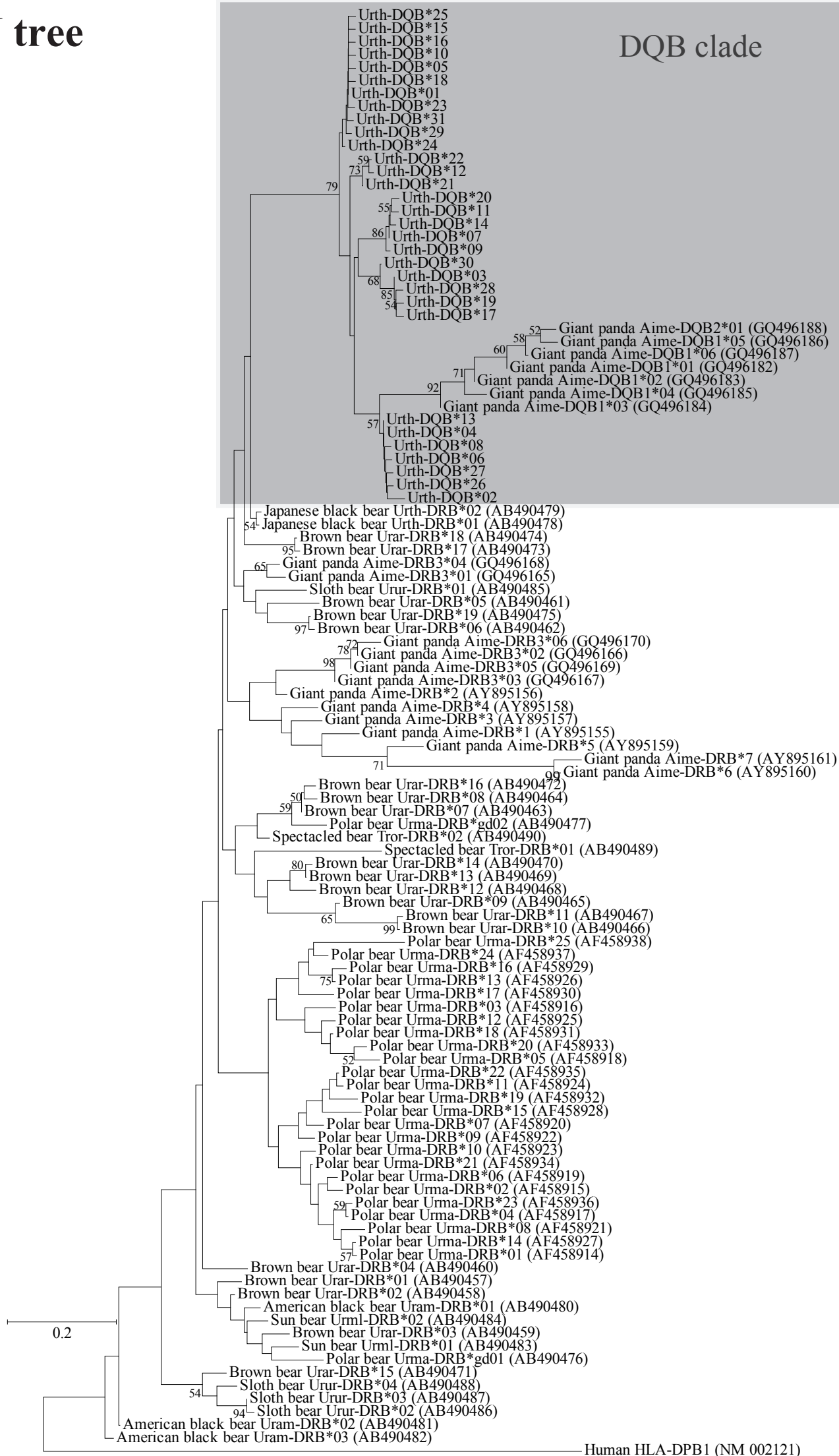

# Bayesian tree

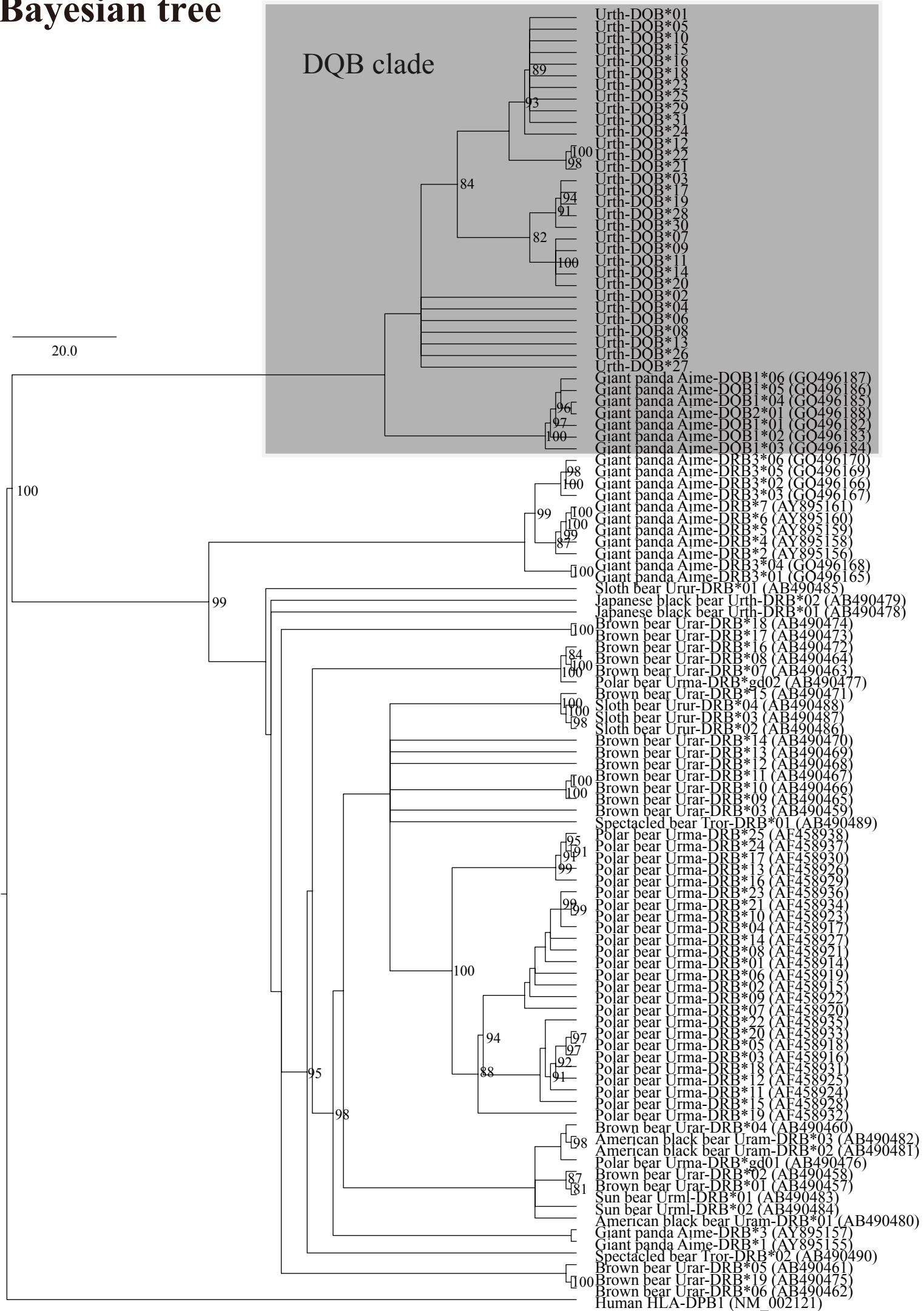

Supplement: Additional file 1 — The NJ and Bayesian trees of ursine DQB and DRB genes. The NJ and Bayesian trees were constructed based on amino acid sequences of partial exon 2 region of ursine DQB or DRB genes. The JTT model with gamma distribution is used for the NJ and Bayesian trees, respectively. Numbers in parentheses are Genbank accession numbers. Only bootstrap values over 50% and posterior probabilities over 80% are shown in the NJ and Bayesian trees, respectively. [file 1471-2148-12-230-S1.pdf]
