# Supplementary material for: MHC class II DQB diversity in the Japanese black bear, Ursus thibetanus japonicus
Source: BMC Evol Biol. 2012 Nov 29;12:230. doi: 10.1186/1471-2148-12-230 (PMC3575356; doi:10.1186/1471-2148-12-230)
Supplement: Additional file 2 — The comparison of nucleotides at the polymorphic sites between uncloned and cloned PCR products. (A) An example that an individual is identified as a heterozygote. The upper figure is the alignment of nucleotide sequences of uncloned and cloned PCR products. The lower figure is the result of direct-sequencing for uncloned PCR product. (B) An example that an individual is not identified as a heterozygote. Such an individual is excluded from analyses of the present study. [file 1471-2148-12-230-S2.pdf]

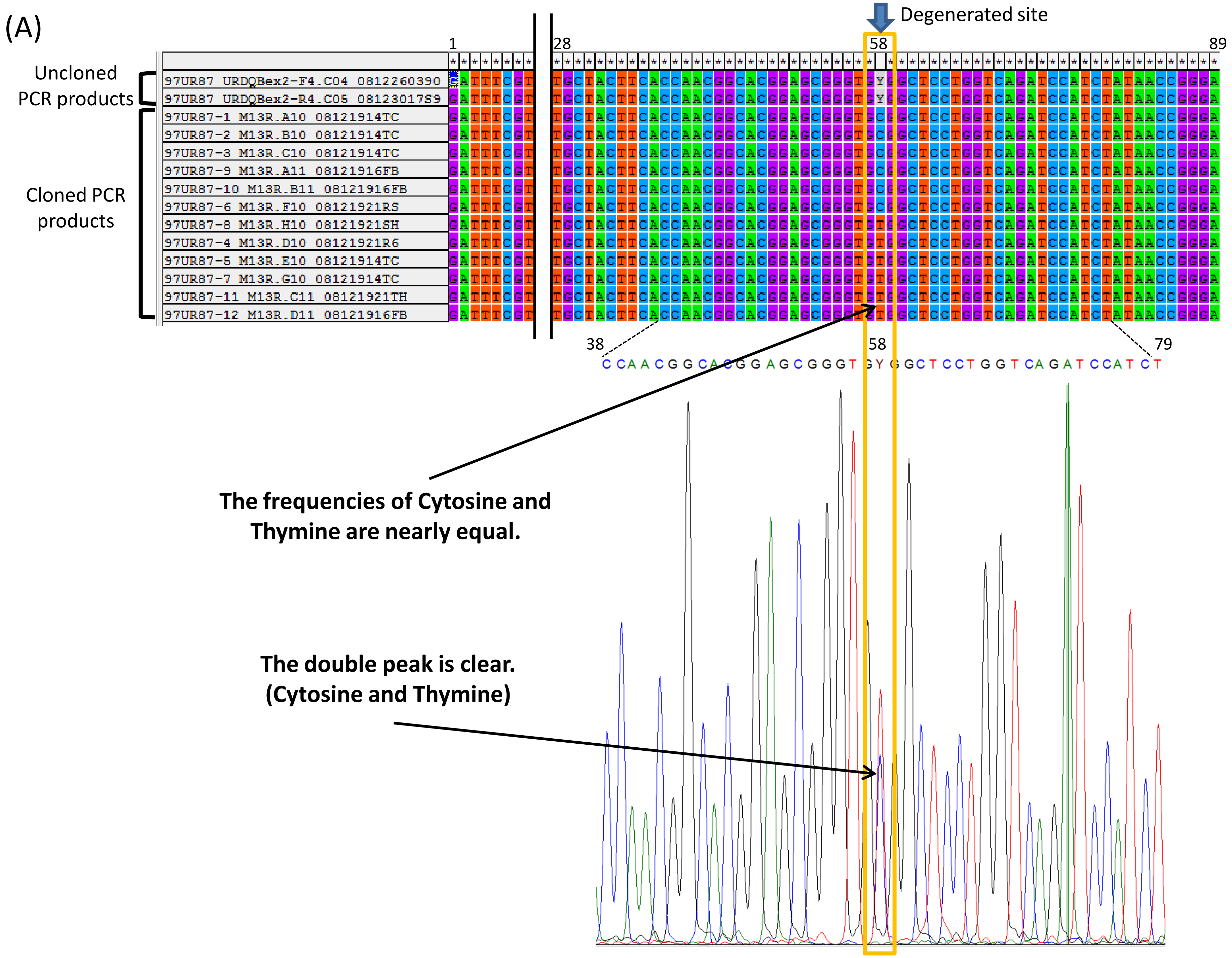
(A)
Degenerated site
1
28
58
89
Uncloned PCR products
Cloned PCR products
97UR87 URDQBex2-F4.C04 0812260390
97UR87 URDQBex2-R4.C05 08123017S9
97UR87-1 M13R.A10 08121914TC
97UR87-2 M13R.B10 08121914TC
97UR87-3 M13R.C10 08121914TC
97UR87-9 M13R.A11 08121916FB
97UR87-10 M13R.B11 08121916FB
97UR87-6 M13R.F10 08121921RS
97UR87-8 M13R.H10 08121921SH
97UR87-4 M13R.D10 08121921R6
97UR87-5 M13R.E10 08121914TC
97UR87-7 M13R.G10 08121914TC
97UR87-11 M13R.C11 08121921TH
97UR87-12 M13R.D11 08121916FB
38
58
79
The frequencies of Cytosine and Thymine are nearly equal.
The double peak is clear. (Cytosine and Thymine)

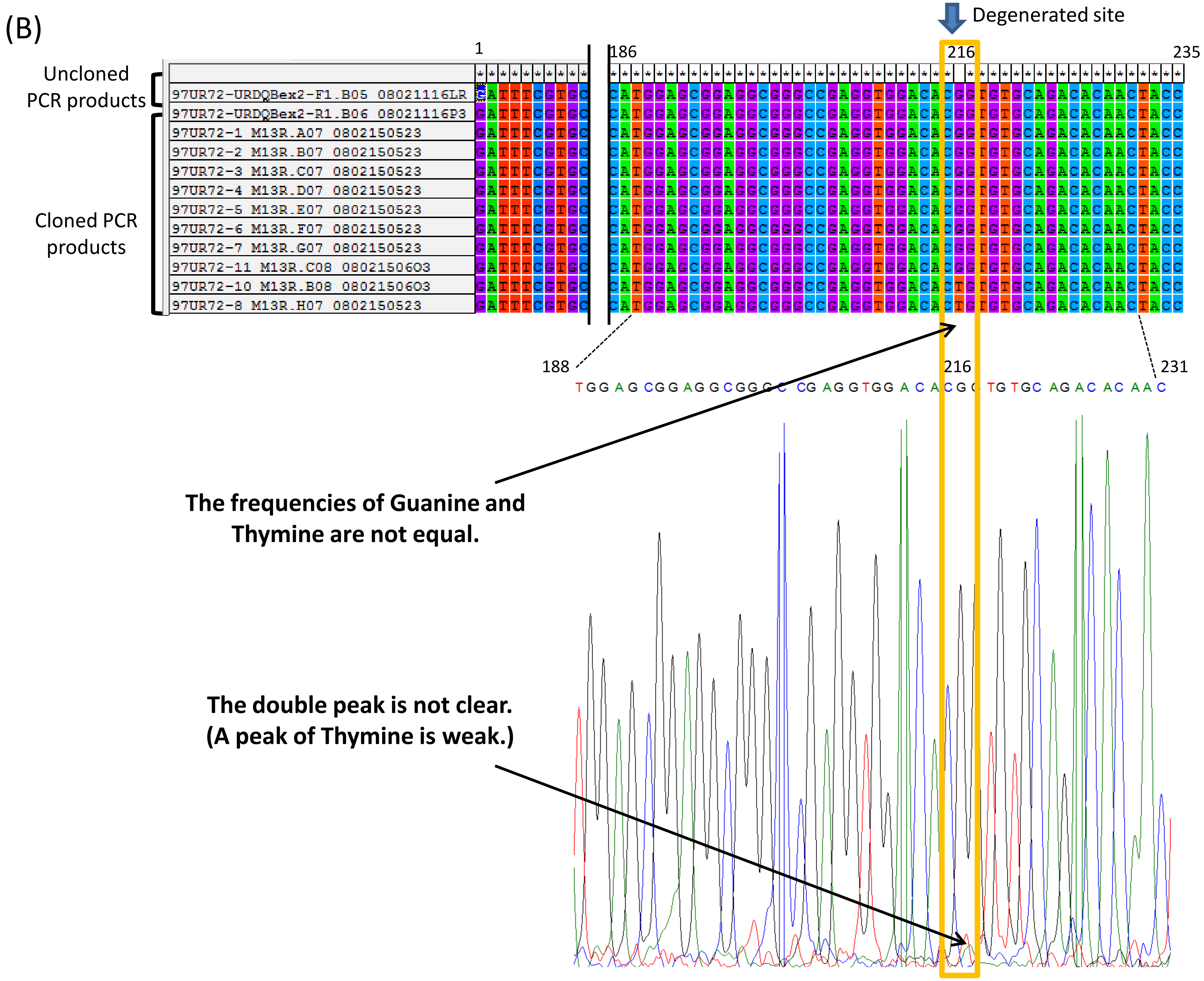
(B)
Degenerated site
Uncloned PCR products
Cloned PCR products
97UR72-URDQBex2-F1.B05 08021116LR
97UR72-URDQBex2-R1.B06 08021116P3
97UR72-1 M13R.A07 0802150523
97UR72-2 M13R.B07 0802150523
97UR72-3 M13R.C07 0802150523
97UR72-4 M13R.D07 0802150523
97UR72-5 M13R.E07 0802150523
97UR72-6 M13R.F07 0802150523
97UR72-7 M13R.G07 0802150523
97UR72-11 M13R.C08 0802150603
97UR72-10 M13R.B08 0802150603
97UR72-8 M13R.H07 0802150523
1
186
216
235
188
216
231
The frequencies of Guanine and Thymine are not equal.
The double peak is not clear. (A peak of Thymine is weak.)
